# Identification of differential genomic DNA Methylation in the hypothalamus of pubertal rat using reduced representation Bisulfite sequencing

**DOI:** 10.1186/s12958-017-0301-2

**Published:** 2017-10-06

**Authors:** Lei Luo, Zhiqiu Yao, Jing Ye, Yuan Tian, Chen Yang, Xiaoxiao Gao, Min Song, Ya Liu, Yunhai Zhang, Yunsheng Li, Xiaorong Zhang, Fugui Fang

**Affiliations:** 1 Anhui Provincial Laboratory of Animal Genetic Resources Protection and Breeding, College of Animal Sciences and Technology, Anhui Agricultural University, 130 Changjiang West Road, Hefei, Anhui 230036 China; 2 Anhui Provincial Laboratory for Local Livestock and Poultry Genetic Resource Conservation and Bio-Breeding, 130 Changjiang West Road, Hefei, Anhui 230036 China; 3 Department of Animal Veterinary Science, College of Animal Science and Technology, Anhui Agricultural University, 130 Changjiang West Road, Hefei, Anhui 230036 China; 4 College of Animal Science and Technology, Anhui Agricultural University, 130 Changjiang West Road, Hefei, Anhui 230036 China

**Keywords:** Epigenetic regulation, DNA methylation, RRBS, Puberty, Methylation profiling

## Abstract

**Background:**

There are many variables affecting the onset of puberty in animals, including genetic, nutritional, and environmental factors. Recent studies suggest that epigenetic regulation, especially DNA methylation, plays a majorrole in the regulation of puberty. However, there have been no reports on DNA methylation of the pubertal genome.

**Methods:**

We investigated DNA methylation in the female rat hypothalamus at prepuberty and puberty using reduced representation bisulfite sequencing technology. The identified genes and signaling pathways exhibiting changes to DNA methylation in pubertal rats were determined by Gene Ontogeny and Kyoto Encyclopedia of Genes and Genomes analysis.

**Results:**

The distribution of the three types of methylated C bases in promoter and CpG island (CGI) regions in the hypothalamus was as follows: 87.79% CG, 3.05% CHG, 9.16% CHH for promoters, and 88.35% CG, 3.21% CHG, 88.35% CHH for CGI in prepubertal rats; and 90.78% CG, 2.13% CHG, 7.09% CHH for promoters, and 88.59% CG, 88.59% CHG, 8.35% CHH for CGI in pubertal animals. CG showed the highest percentage of methylation, and was the highest methylation state in CGI. Compared to prepubertal hyoyhalamus samples, we identified ten genes with altered methylation in promoter regions in the pubertal hypothalamus samples, and 43 genes with altered methylation in the CGI. Changes in DNA methylation were found in gonadotropin-releasing hormone signaling pathways, and the oocyte meiosis pathway.

**Conclusion:**

Our results demonstrate changes in DNA methylation occur in female rats from prepuberty to puberty suggestng DNA methylation may play a crucial role in the regulation of puberty onset. This study provides essential information for future studies on the role of epigenetics in the regulation of puberty.

**Electronic supplementary material:**

The online version of this article (10.1186/s12958-017-0301-2) contains supplementary material, which is available to authorized users.

## Background

Puberty is defined as the time of transition from childhood to adulthood [1]. Physiologically, puberty is the point at which an animal is first capable of reproducing sexually; in the female, this is the first occurrence of menstruation and in the male the testes begin to undergo spermatogenesis. In humans, early and rapidly progressive puberty is harmful [2–5]. In contrast, domesticated animals are bred for early puberty [6, 7]. In animal production, breeding of early puberty livestock can save feeding costs and improve the utilization of animal, while it can also shorten the generation interval of fine animals and accelerate the genetic breeding process [8, 9].

Pubertal timing is influenced by complex interactions involving genetic [10], nutritional [11], environmental [12], and (in humans) socioeconomic factors [13]. The traditional view is that hypothalamic gonadotropin-releasing hormone (GnRH) neurons are sensitive to negative feedback by the gonadal hormones before puberty. Therefore, due to inhibition by gonadal hormones, a low amplitude and frequency, pulsed secretion of GnRH is observed from birth to puberty onset. The onset of puberty begins with the release of this inhibitory mechanism in the hypothalamus, and re-activation of a high amplitude and frequency GnRH secretion pulse. In turn, GnRH acts on GnRH receptors in anterior pituitary follicle stimulating hormone (FSH) and luteinizing hormone (LH) secretory cells, thereby promoting secretion of LH and FSH. In turn, LH and FSH act on the gonads, stimulating the secretion of testosterone in males or estradiol secretion in females, thereby promoting the rapid development of reproductive organs and sexual characteristics.

A specific switch may exist to control hypothalamic GnRH secretion, and induce puberty onset; however, its precise mechanism remains to be determined. Evidence suggests the initiation of puberty requires the coordinated activity of genes organized into functional networks [14–16]. A large number of genes involved in neuroendocine regulation control of the onset of puberty [17, 18]. Elks et al. reported that more than 30 gene variants were associated with age at menarche in humans [19], supporting the claim to that no one gene controls the onset of puberty [20]. At present, important genes in the hypothalamus related to the onset of puberty include the *Kiss-1/GPR54* system [21, 22], NPY [23, 24], Leptin [25, 26], *LIN28B/let-7* [27] and *NKB* [28–30]. Polygenic inheritance is the basis of the onset of puberty [20, 31, 32], and is closely related to hypothalamic correlation factors, transcription and changes in the expression levels of receptor genes. However, how these puberty-related genes are activated, and how the dynamic regulation of gene expression is coordinated during the onset of puberty, remains unclear.

Epigenetic regulation of gene expression is now recognized as an important player in the complexity of DNA and histone modification patterns, leading to huge differences in cell phenotype and function [32]. Epigenetic regulation is sensitive to stimuli, and cells are able to adapt to their environment through these modifications. However, the modification of these complex patterns has not been fully explained. The neuroendocrine system is known to have a high degree of sensitivity to environmental changes, so in recent years, epigenetic control of neuroendocrine function has become a focus of much research. It has been confirmed that DNA methylation plays an important role in the puberty onset in rats [32–34]. In particular, hypothalamic expression of two key genes (Eed and Cbx7) decreased and methylation of their promoters increased before puberty; inhibiting DNA methylation blocked both events and resulted in pubertal failure [32].

Despite these data, little information is available on DNA methylation profiling of the rat pubertal genome. In this research, we performed reduced representation bisulfite sequencing (RRBS), a cost-efficient method for genome-scale DNA methylation analysis [35]. The aim of the current study was to investigate the DNA methylation profile of the hypothalamic genome in the pubertal rat at a single base resolution.

## Methods

### Animals

For the first 19 days after pairing, litters were assessed daily, and the day of birth was considered postnatal day 1. The animals were weaned on day 21. The rats were kept under standard conditions (12:12 h light-dark cycle with lights on between 06:00 and 18:00 h; temperature, 22 ± 1 °C; rat chow and water provided ad libitum; cage size: L × W × H,46.5 × 34.5 × 20 cm). Prepubertal samples were collected at postnatal day 25 and pubertal samples were collected on the day of vaginal opening (postnatal day 36.2 ± 0.58) at the stage of estrus.

### Sample preparation and genomic DNA isolation

**Fig. 1:**
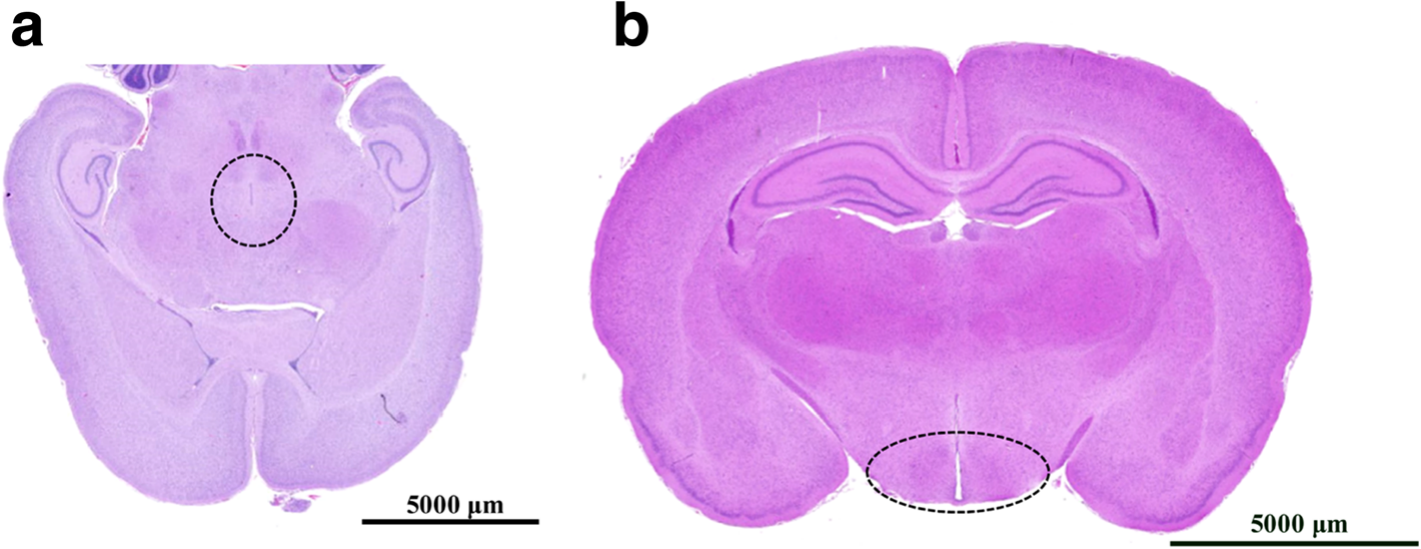
The location of the hypothalamus in the brain.Representative horizontal (**a**) and vertical (**b**) images of histological section of a rat brain. The black circle indicates the position of the hypothalamus

Female rats were sacrificed using cervical dislocation. The hypothalamus was dissected using the tuber cinereum as the ventral landmark for cuts to remove the frontal lobe and lateral and posterior portions of the brain. The cortex was peeled away from the remaining ventral brain piece containing the hypothalamus [36, 37]. The position of the hypothalamus was indicated by HE staining (Fig. 1a,b). Samples were frozen in liquid nitrogen immediately and stored at −80 °C until use.

The hypothalami from five rats each in the prepubertal of pubertal groups were mixed together and genomic DNA was extracted. Genomic DNA was isolated using AxyPrep™ Multisource Genomic DNA Miniprep Kit (Corning, AP-MN-MS-GDNA-50G), according to the manufacturer’s protocol. The quality of the genomic DNA was checked by the NANODROP 2000 UV DNA Analyzer of which the minimum detection sample volume is 0.5 μl and the wavelength range is 190-840 nm. (Thermo scientific).

### Library construction and sequencing

The library construction and sequencing was performed at BGI-Shenzhen (Shenzhen, China). Briefly, at least 3 μg of total genomic DNA from each sample was digested with MspI (NEB, R0106T). The digestion was followed by DNA end-repair, the addition of a single A nucleotide, and multiplexed adapter ligation. The DNA samples were purified by agarose gel electrophoresis to isolate 40–220 bp long fragments. We then used the ZYMO EZ DNA Methylation-Gold™ kit (ZYMO RESEARCH, D5006) to get bisulfite conversion, and amplified the products by PCR. The quality of library was analyzed by the Agilent 2100 Bioanalyzer (Agilent Technologies), of which the DNA size analysis range is 25–12,000 bp and quantified by the ABI StepOnePlus Real time-PCR System (Thermo Fisher). The resulting fragments were sequenced on an Illumina Hiseq2000 analyzer (Illumina). To determine the location of each clean read before doing methylation analysis, we used Bsmap 2.74 to compare with the reference genome (*R. norvegicus*, assembly Rno-6.0, http://www.ncbi.nlm.nih.gov/assembly/GCA_0000018 95.4).

### RRBS data analysis

RRBS utilizes the restriction enzyme Mspl to digest the entire genome, and allows for enrichment of sequences with relatively high CpG content (i.e., promoter and CpG islands [CGI] regions). These fragments are sequenced after bisulfite treatment (to convert unmethylated cytosine), and analysis of the methylation status of each base site is performed [38, 39]. Although only restriction fragments are sequenced, they mainly cover CpG-rich regions, making it possible to judge the state of methylation of the whole genome from RRBS results.

We analyzed the numbers of promoters and CGI (CG, CHG, CHH), cytosine site coverage analysis, the cumulative distribution of cytosine effective sequencing depth and compared the theoretical coverage and the actual depth of coverage on CG sites. Methylation analysis of promoter and CGI regions was done through the distribution ratio of CG, CHG and CHH in methylated C bases, the average methylation level of C bases and the distribution of different types of methylation levels. Regional methylation profiling was calculated by the association between methylation level and CpG density in a specific area, and the CpG density was defined for each CpG site within a window of 200 bp. Analysis of differentially methylated regions (DMRs) using sliding window method was performed to calculate the DMRs between prepubertal and pubertal samples.

### Functional annotation of DMRs

DMRs were used for functional annotation, including Gene Ontology (GO) and Kyoto Encyclopedia of Genes and Genomes (KEGG) pathway analysis. GO analysis is the international standard for gene function classification, which fully describes the properties of genes and gene products in organisms [40]. In brief, all the identified genes are mapped to each term in GO database, gene numbers for each term are calculated, and then a hyper geometric test is used to find significantly enriched GO terms in DMRs compared to the reference gene background and obtained a *p*-value. The Bonferroni Correction for the p-value was used to obtain a corrected p-value. Differential expression is determined and the corrected p-value (<0.05) is the threshold for significant differences in gene expression within each GO term, and allows for identification of differentially expressed genes in pathways of major biological functions. The gene sequences were then compared to the NCBI non-redundant protein database by using BLAST, and the results were annotated to GO using Blast2 GO.

KEGG is the major public databases analysis of pathway [41] that can be used to better understand the biological function of genes. Significant enrichment of pathways is determined by hypergeometric test identifying the significantly altered pathways in differentially expressed genes after comparing to the entire genome. The Q Value is set at <0.05 as the threshold for significance, and gene sequences were annotated to KEGG databases by BLAST. Rich Factor analysis was then used to determine the ratio of the number of the genes located in pathways of differentially expressed genes and in all annotated genes; the greater the Rich Factor, the greater the degree of enrichment.

### Isolation of total RNA and quantitative real-time PCR

Total RNA was isolated using a Transzol Up Plus RNA Kit (Transgen Biotech, Beijing, China) following the manufacturer’s instructions. RNA degradation and contamination was monitored on 1% agaroes gels. RNA purity was detected using the NanoDrop 2000c spectrophotometer (Thermo Scientific, IL, USA). Reverse transcription was carried by HiScript® QRT SuperMix for qPCR(+gDNA wiper)(R123–1, Vazyme, Nanjing, China). Real-time PCR was performed using a StepOnePlus Real-time PCR Instrument (Applied Biosystems) in a 15 reaction system as described in the instructions of AceQTM qPCR SYBR® Green Master Mix (Q121–02, Vazyme, Nanjing, China). Reaction were incubated in a 96-well optical plate (Applied Biosystems) at 95 °C for 10 min, followed by 40 cycles of 95 °C for 30s, 60°Cfor 30s, and 72 °C for 30s. Each sample was run in triplicate. Primer sequences were synthesized by Sangon Biotech (Shanghai, China) on the basis of the gene sequences obtained from the NCBI database. Primer sequences are lised in Table 1. Gene expression was quantified relative to β-actin expression using the 2^-ΔΔCT^ method.

**Table 1:** Primers used in real-time PCR

Gene	Forward primer, 5′-3′	Reverse primer, 5′-3′	Product, bp
*Itpr2*	ATCAGATGCCTGCCTCATCG	GTTCTGGGAGCTGAATGGCT	115
*Gnas*	CAGCCCGAGCAAGAACCTTT	CGGGGATGGGCTCATTGTTA	105
*β-actin*	CCCATCTATGAGGGTTACGC	TTTAATTGTCACGCACGATTTC	150

## Results

### Coverage analysis of promoter and CGI regions

After library construction, alignments were compared to the reference genome sequences. We had 248,727,316 and 244,131,868 clean reads from the prepubertal and pubertal samples, respectively (Additional file 1: Table S1). The enzyme rate which is the the unique reads containing enzyme loci percentage of total unique reads was 77.19% and 75.64%, and the bisulfite conversion rate was 99.32% and 99.39%, respectively. Our RRBS covered most promoters, although not all of the genome; we list the information covered, including the total number covered in the genome, the theoretical value in enzyme fragments and the actual value covered by at least 5 CGs in Additional file 1: Table S2. The total number of promoters covered was 18,845, the theoretical value (target region) was 174, and the actual coverage value was 66 in prepubertal and 64 in pubertal samples (Additional file 1: Table S2).

For CGI, the theoretical value indicates location of cytosine in the theoretical enzyme cutting region, and the experimental value is the actual number of cytosines covered by our sequencing reads. We investigated the numbers of each cytosine type identified (CG, CHG and CHH; H represents non-G base, hereinafter inclusive). In the promoter regions, the actual coverage rate of C sites (CG, CHG, CHH), was 35.69% CG, 18.61% CHG and 13.56% CHH in prepubertal samples, but was 33.66% CG, 16.96% CHG and 12.14% CHH in pubertal samples (Table 2). In CGI in prepubertal samples, the actual distribution of C sites (CG, CHG, CHH) was 52.17% CG, 53.76% CHG and 50.17% of CHH; in pubertal samples, the distribution was 51.36% CG, 52.92% CHG and 49.32% CHH (Table 2). The number of C sites in the genome, the number of theoretical fragments of C sites, and the number of the actual coverage sites are shown in Table 2.

**Table 2:** Cytosine distribution

		Promoter			CGI		
		CG	CHG	CHH	CG	CHG	CHH
Genome		13,714	34,767	107,908	2,605,360	2,108,472	4,111,032
Target region		13,714	34,769	107,924	2,605,322	2,108,514	4,111,734
Prepuberty	Number	4894	6472	14,635	1,359,226	1,133,535	2,062,809
	Rate (%)	35.69	18.61	13.56	52.17	53.76	50.17
Puberty	Number	4616	5898	13,106	1,338,206	1,115,821	2,027,863
	Rate (%)	33.66	16.96	12.14	51.36	52.92	49.32

### Methylation analysis of promoter and CGI regions

**Fig. 2:**
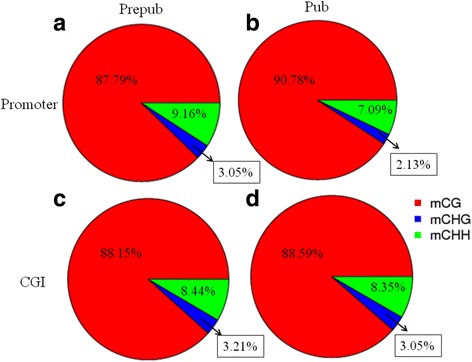
Different methylation at C bases in the hypothalamus of prepubertal and pubertal rats. Methylation at CG, CHG, and CHH in promoter regions in (**a**) prepubertal samples and (**b**) pubertal samples, as well as in CGI in (**c**) prepubertal samples and (**d**) pubertal samples. mCG, mCHG, and mCHH represent the methylated forms of CG, CHG and CHH

We calculated the ratio of the three types of methylated cytosine (mCG, mCHG, mCHH) in promoters and CGI. We found that compared with the prepubertal samples, in promoter regions from pubertal samples, the proportion of mCG increased 2.99%, while mCHG and mCHH decreased 0.92% and 2.07% (Fig. 2a and c). The same result was seen in CGI, with the proportion of mCG increased 0.24%, and mCHG and mCHH decreased 0.16% and 0.19% (Fig. 2b and d).

**Fig. 3:**
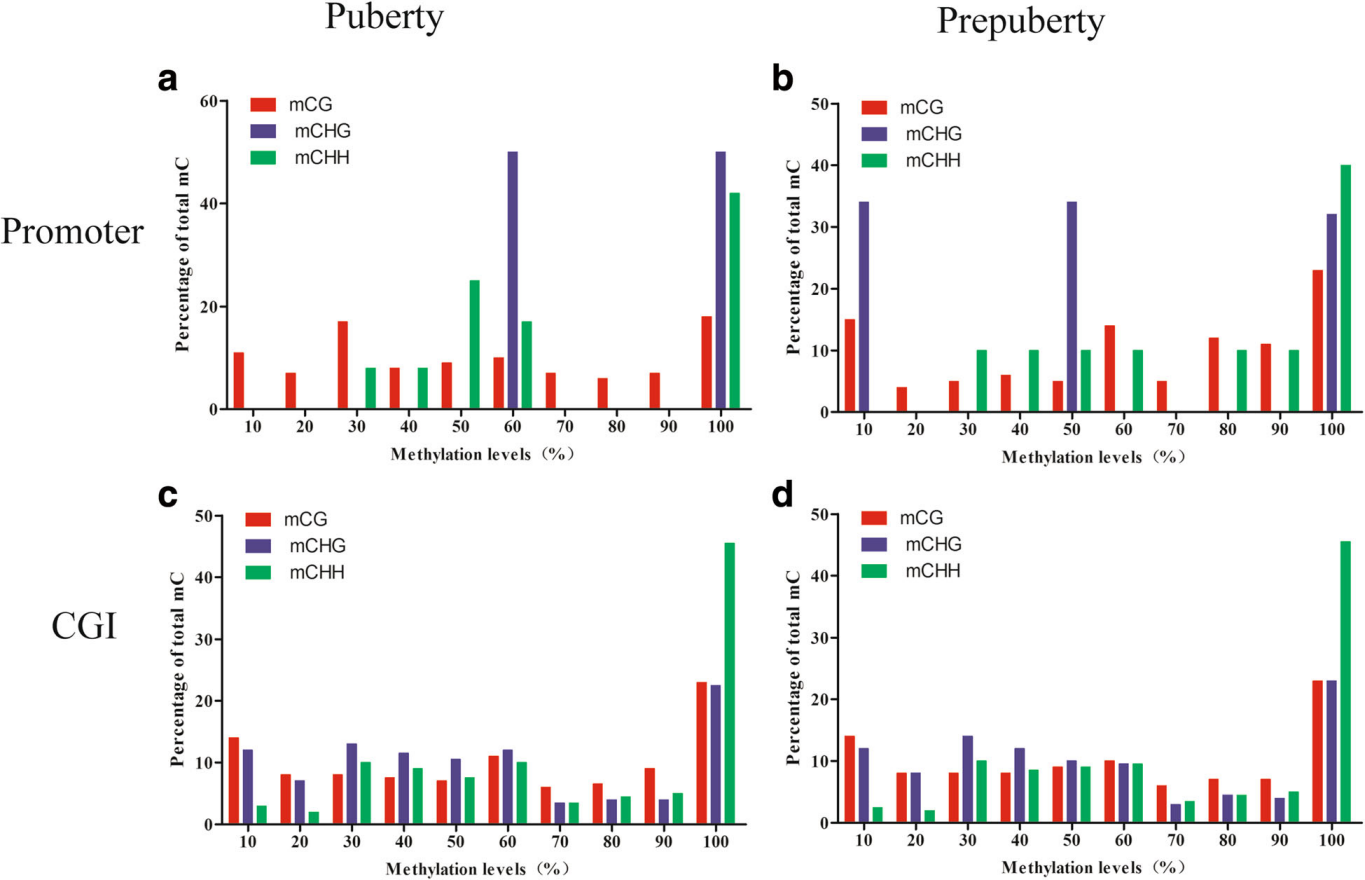
Distribution of mC bases in the hypothalamus of prepubertal and pubertal rats. Distribution of methylation at mCG, mCHG, and mCHH in promoter regions in (**a**) prepubertal samples and (**b**) pubertal samples, as well as in CGI in (**c**) prepubertal samples and (**d**) pubertal samples. The x-axis indicates methylation level, and the y-axis shows the percentage of all mC at a certain level of methylation. mCG, mCHG, and mCHH are represented by the red, blue, and green lines, respectively

We then analyzed the average methylation level of all C bases. CG had the highest average level of methylation in both prepubertal and pubertal samples, whether in the promoter or CGI. Investigating further, we found that mCHH had the highest average level of methylation in promoters in both groups. However, in CGI, mCHH had the highest average level of methylation in the prepubertal samples, while mCG as the highest in the pubertal samples group (Table 3). Finally, we analyzed the percentage of methylation of methylated cytosines in each sequence context. In promoter regions, mCG was distributed evenly in pubertal samples. The distribution of mCHH was almost zero in low methylation state (<20% of the distribution), but was more than 40% of mCHH in high methylation states (>90%). mCHG were mainly distributed at approximately 60% and approximately 100% high methylation levels in prepubertal samples, and in pubertal samples were mainly distributed in 10%, 50% and >90% of the three methylation status (Fig. 3a and c). The distribution of methylation level in CGI was different from the distribution in promoters: mCG, mCHG, mCHH methylation levels were highly distributed (>90%), and especially for mCHH, nearly 45% were hypermethylated (Fig. 3b and d).

**Table 3:** Average methylation levels of C and mC

Sample	Element	C, CG, CHG, CHH average methylation levels				mC, mCG, mCHG, mCHH average methylation levels			
		C	CG	CHG	CHH	mC	mCG	mCHG	mCHH
Prepuberty	Promoter	1.530	2.795	0.667	1.220	28.037	19.953	87.879	91.921
	CGI	3.156	6.462	0.891	1.906	27.397	26.916	10.944	40.440
Puberty	Promoter	1.531	2.929	0.604	1.167	24.924	18.810	18.012	92.718
	CGI	3.825	8.346	0.893	1.962	27.888	31.651	8.018	26.812

### Regional methylation profiling

**Fig. 4:**
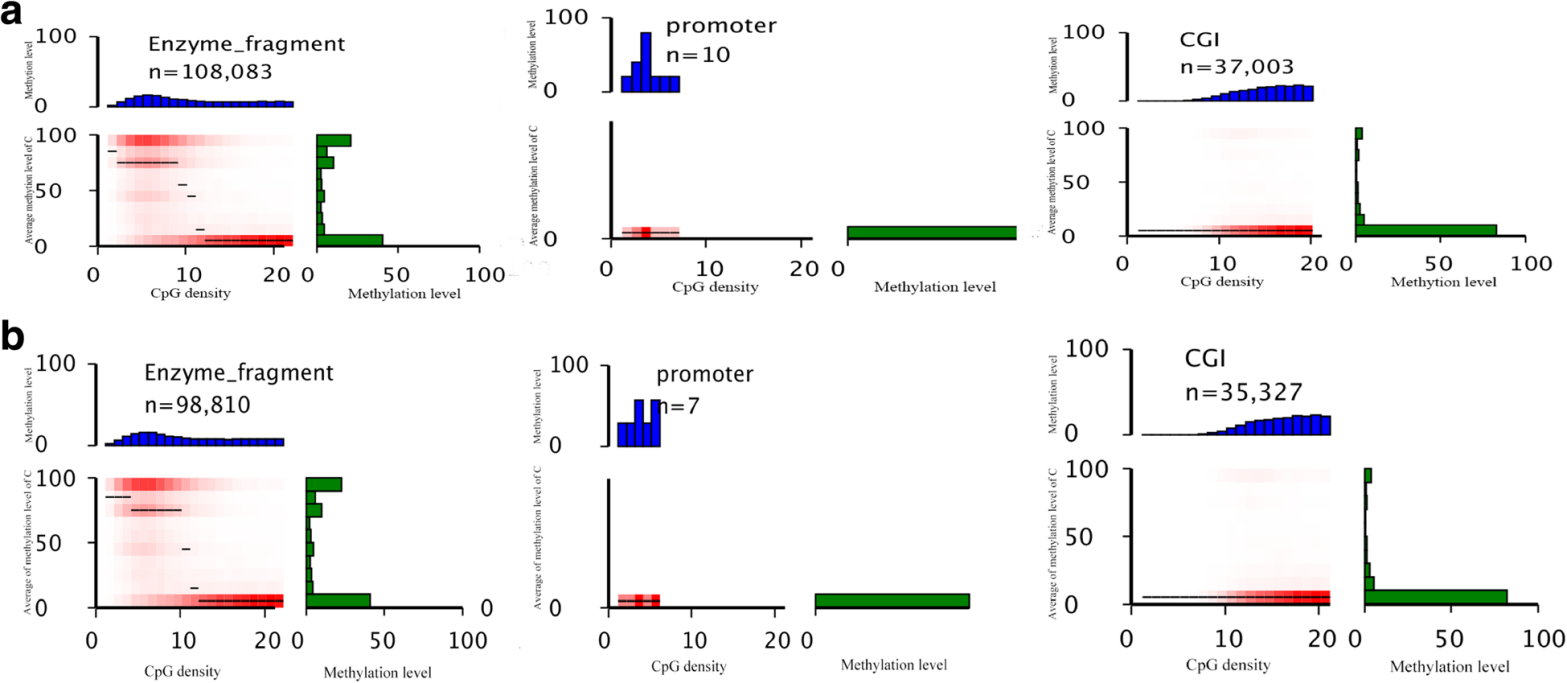
Heat maps showing methylation distribution and CpG density distribution. Heat maps of (**a**) Prepubertal and (**b**) pubertal samples. n refers to the number of analyzed CpGs (per-strand depth ≥ 4) within that feature. CpG density (x-axis) is defined as the number of CpG dinucleotides within the 200 bp window. Methylation level (y-axis) is defined as average methylation level of cytosines in CpGs. The thin black lines within each heat map denote the median methylation level of CpGs at the given local density. The red gradient indicates abundance of CpGs that fall into bins of given methylation levels and CpG densities. The blue bar charts above each heat map show the distribution of CpG densities, projected onto the x-axis of the heat maps. The green bar charts to the right of the heat maps show the distribution of methylation levels, projected onto the y-axis of the heat maps

We used heat mapping to represent methylation distribution, CpG density distribution and the relationship between methylation and density. In prepubertal samples, there were 108,083 restriction fragments, 10 fragments of promoters, and 37,003 fragment of CGI were identified in this analysis (Fig. 4a). In all enzyme fragments, both in prepubertal and pubertal samples, methylation levels were dichotomized: high methylation levels with low CpG density and low methylation levels with high CpG density. CpG density was generally low in promoters and high in CGIs; both regions showed low levels of methylation (Fig. 4a and b). Hypermethylated and hypomethylated C in enzyme fragments indicated a higher proportion than others while the majority proportion C was in low methylation levels in promoter and CGI. The heat map of CHG and CHH showed similar results.

### Identification of differentially methylated regions and bioinformatics analysis

We identified DMRs between prepubertal and pubertal samples, as shown in Table 4. The 23 chromosomes investigated showed a total of 152 DMRs. The identity of the differentially methylated genes are shown in Table 5; if the log2 ratio value is positive, that indicates upregulation, while a negative log2 ratio value indicates downregulation. In promoter regions, there were eight differentially methylated genes that were upregulated, and two were downregulated. In CGI regions, 42 differentially methylated genes were upregulated, and 10 were downregulated.

**Table 4:** Methylation regional analysis

Chromosom	1	2	3	4	5	6	7	8	9	10	11	12	13	14	15	16	17	18	19	20	X	Y	M
Numbers of DMR	12	5	9	6	13	5	16	6	8	10	4	6	2	7	6	3	4	5	2	5	18	0	0
Length of DMR	3819	1953	2399	1633	3996	1343	4751	1651	2271	4019	1152	2225	547	1611	1672	931	1628	1267	769	1396	4279	0	0

**Table 5:** Gene with Differentiation Methylated Regions across puberty

	Promoter	CGI
Upregulation gene ID(Log2 Ratio)		299,907(20), 117,261(3.369), 302,424(2.585),315,059(2.563),361,576(2.478), 287,534(2.17),310,926(1.939),
		24,896(1.893), 306,761(1.893),297,604(1.728), 24,944(1.585), 116,727(1.497),116,728(1.497), 29,714(1.447),
	366,270(2.433),24,896(1.893),	29,589(1.441), 363,256(1.396), 24,413(1.379), 288,271(1.344),25,177(1.322),308,022(1.287),
	29,361(1.485),288,271(1.344),	681,377(1.278),369,017(1.263), 280,670(1.239), 29,185(1.233), 246,299(1.182),
	25,177(1.322),83,533(1.322),	498,194(1.162), 314,694(1.158), 362,945(1.138), 64,553(1.129), 300,653(1.115), 79,219(1.112), 303,678(1.105),
	314,746(1.234), 299,266(1.155)	64,445(1.064), 313,022(1.059), 317,346(1.044), 29,616(1.034), 362,456(1.034), 84,573(1.033), 81,678(1.019), 301,056(1.016), 24,409(1), 85,251(1)
Downregulation gene ID(Log2 Ratio)	317,425(−2.17),501,559(−1.396)	94,268(−3.7), 293,976(−2.28), 313,596(−1.632), 361,833(−1.423), 287,709(−1.415), 501,559(−1.396), 360,973(−1.363), 297,096(−1.268), 306,141(306141), 308,099(−1.23)

Statistical significance of GO and KEGG enrichment analysis to identify differentially methylated genes was evaluated by the hypergeometric tes. GO functional analysis indicated that differentially methylated genes in promoter regions were enriched in 106 major functional groups. In promoter regions, six differentially methylated genes were found involved in the 14 cellular component groups (Additional file 1: Table S3), six differentially methylated gene were involved in 13 molecular function groups (Additional file 1: Table S4), and seven differentially methylated genes were involved in 79 biological process groups (Additional file 1: Table S5).

**Fig. 5:**
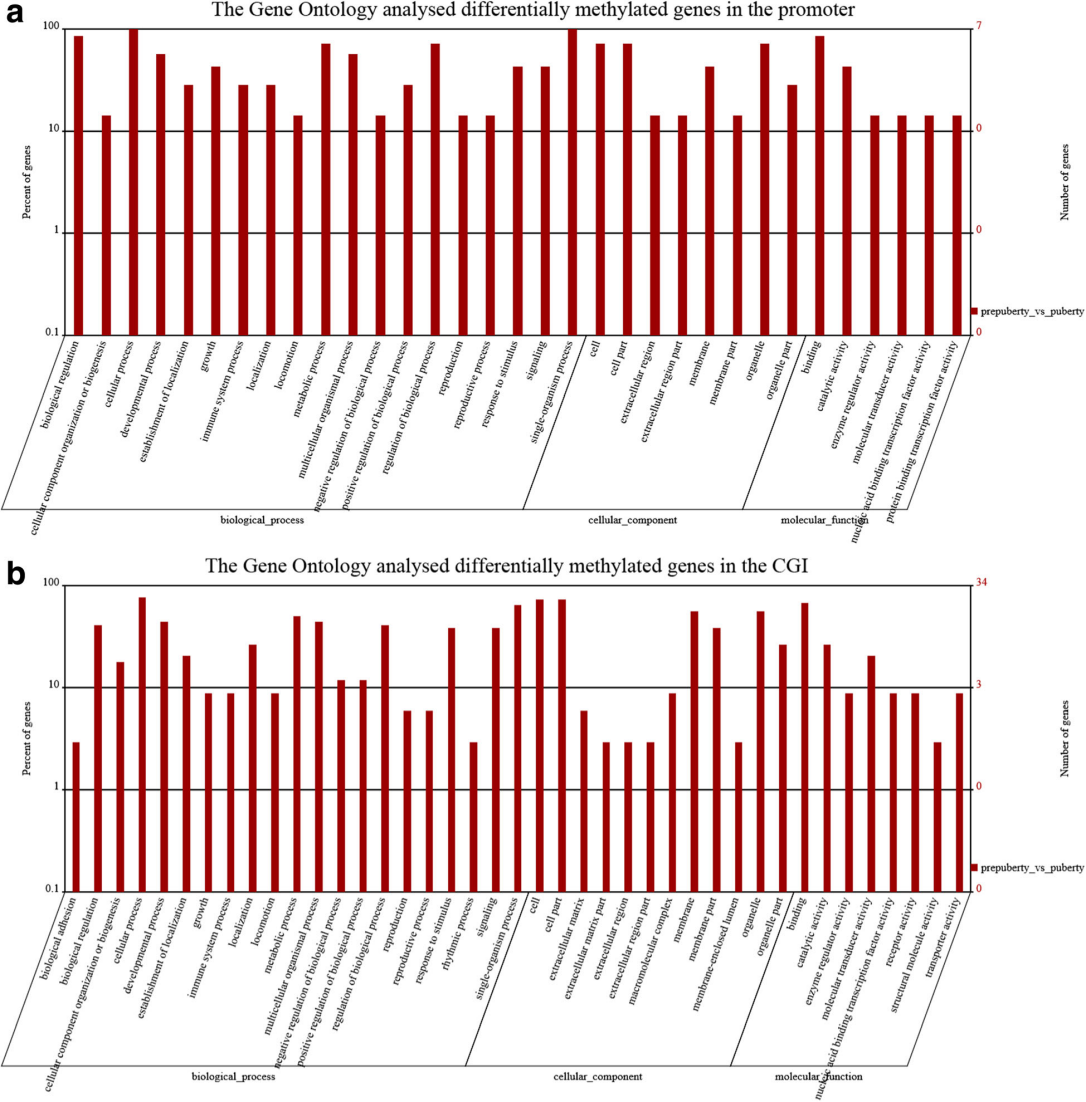
The Gene Ontology functional analysis of prepubertal and pubertal rat hypothalamus. Gene Ontology (GO) analysis of differentially methylated genes between the hypothalamic genome of prepubertal and pubertal rats in (**a**) the promoter and (**b**) the CGI regions. The y-axes on the left represents the percentage of genes and the one on the right is the number of genes involved in different functional groups. The x-axis is the name of the functional groups within the three GO terms, cellular component, molecular function and biological process

The numbers of differentially methylated genes were identified in the functional groups of single-organism process, biological regulation, cellular process in biological process and binding functional groups in molecular function (Fig. 5a). In CGI, differentially methylated genes were enriched in 348 functional groups; there were 31 differentially methylated genes involved in the 47 cellular component groups (Additional file 1: Table S6), 28 differentially methylated genes involved in 63 molecular function groups (Additional file 1: Table S7), and 28 differentially methylated genes involved in 238 biological processes (Additional file 1: Table S8). Cellular process, single-organism process, cell, cell part and binding functional groups occupied the dominant positions (Fig. 5b). This evidence suggests that differentially methylated genes are involved in a variety of biological processes.

**Fig. 6:**
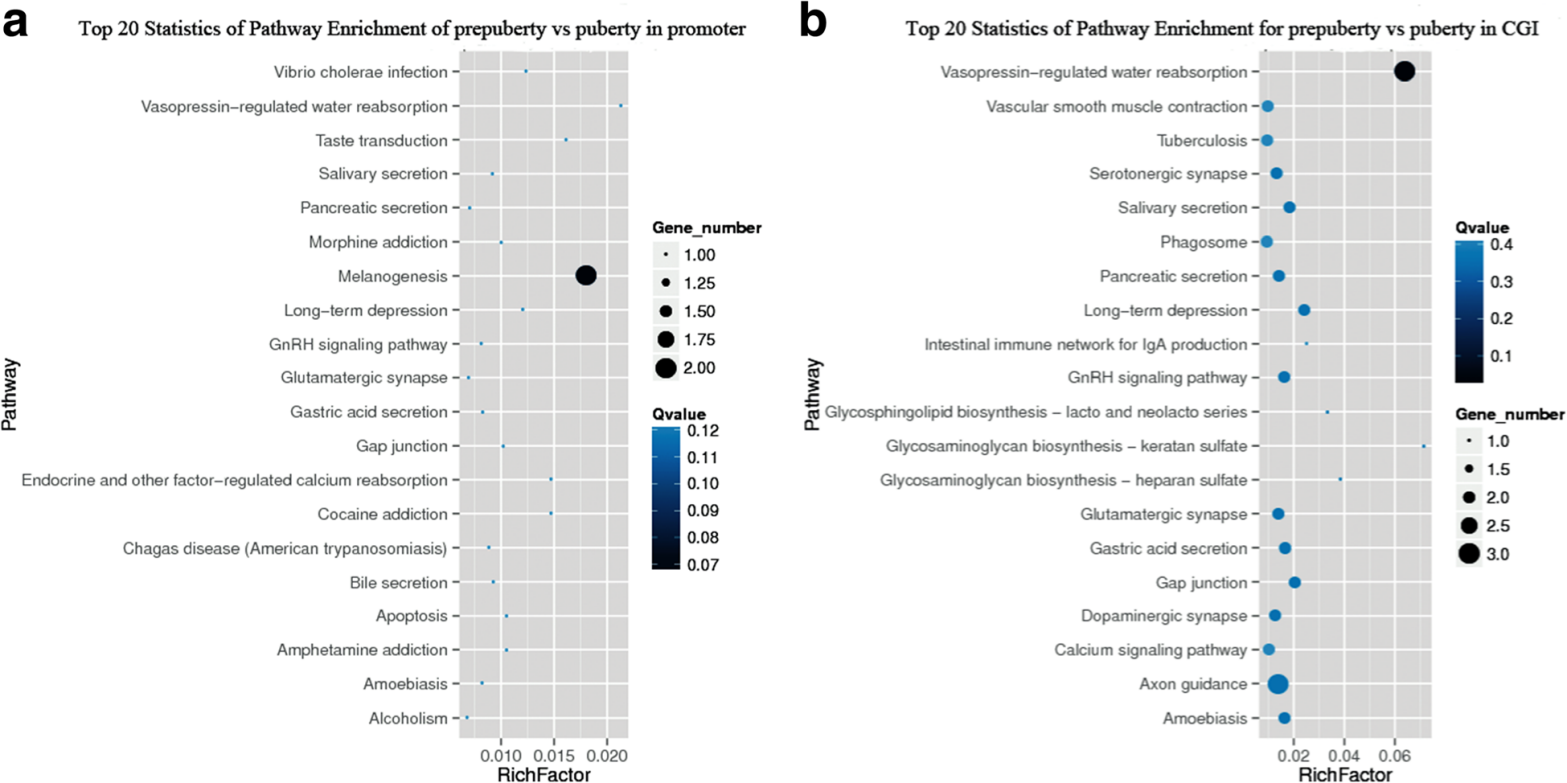
Top 20 enriched pathways in the hypothalamic genome of prepubertal and pubertal rats. KEGG pathway analysis showing the top 20 enriched pathways in the (**a**) promoter and (**b**) CGI regions of differentially methylated genes from the prepubertal and pubertal rats. Rich factor relates to the ratio between the number of genes enriched in the pathway and annotated in differentially methylated regions (DMRs). Rich factor and enrichment are positively correlated. The Q value is the *P* value that was corrected after multiple hypothesis testing. Q value and enrichment are in a negatively correlated

**Fig. 7:**
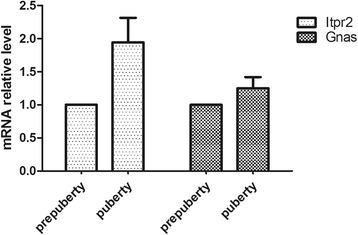
Expression of the *Itpr2* and *Gnas* in prepubertal and pubertal samples by quantitative PCR

Using KEGG Pathway analysis, we found that differentially methylated genes were involved in 25 (Additional file 1: Table S9) and 57(Additional file 1: Table S10) pathways, respectively in promoter and CGI. The pathways with the largest number of differentially methylated genes in the promoter region were the melanogenesis pathways, and the pathway with the largest Rich Factor value was the vasopressin-regulated water reabsorption pathway (Fig. 5a). Rich factor relates to the ratio between the number of genes enriched in pathway and annotated in DMR. Rich factor and enrichment are positively correlated. The GnRH signaling pathway also had a high degree of enrichment (Fig. 6a). The pathway with the largest number of differentially methylated genes in CGI was the vasopressin-regulated water reabsorption pathway, and the highest Rich Factor value pathway was the glycosaminoglycan biosynthesis-keratin sulfate (Fig. 6b). This analysis identified the GnRH signaling pathway and the oocyte meiosis pathway (Fig 5b). Thus, differentially methylated genes were shown to be extensively involved in many aspects of cell metabolism. Interestingly, the expression of *Gnas* and *Itpr2* whcih were enriched in the GnRH signaling pathways and the oocyte meiosis pathways were detected and showed no variation from prepuberty to puberty (Fig. 7).

## Discussion

In this study, we found methylation predominated at CG sites, with differential methylation levels across the CGI versus promoter sites. We also found an increase in the proportion of methylated Cs in CG sites (over CHG and CHH sites) in promoter regions in pubertal hypothalamus samples, and a reduction in the methylation levels at mCHG sites in promoter regions. Genes that were differentially regulated during puberty were involved in the vasopressin-regulated water reabsorption and GnRH signaling pathways. The results presented here provide the initial targets for future studies of epigenetic regulation in the onset of puberty, and enhance our understanding of the epigenetic mechanisms involved.

Our data showed that the highest proportion of methylation occurs on CG, which conforms to the known characteristics of mammal methylation. There are differences in DNA methylation between different species, and in different tissues from the same species. For example, transposon elements and promoter methylation level is higher than in the gene and the coding sequence [39, 42]. In plants, Ziller et al. confirmed that DNA methylation states differ in different plant organs and different developmental stages of the same organ [43]. Different types of C site methylation (mCG, mCHG, mCHH) occur in the genome in different proportions; the ratio of each type of mC site in all the mC sites reflects the characteristics of methylation in a particular species and period to a certain extent.

In this study, we examined the proportion of the three types of C bases (mCG, mCHG, mCHH) in the promoter and CGI regions. Comparing pubertal to prepubertal samples, the ratio of CG increased 2.99%, while CHG and CHH decreased 0.29% and 2.07% respectively, in promoter regions. The ratio of CG increase 0.24% and the ratio of CHG and CHH decrease 0.09% and 0.16% respectively in CGI regions. Recent Recent studies have shown non-CpG methylation (mCHG, mCHH) plays a role in transcriptional repression [44, 45]. In this study, the ratio of CG increased, while CHG and CHH decreased in both the promoter regions and the CGI regions. Those changes may be related to the initiation of transcription of some genes during puberty onset, although whether these changes can be used to monitor puberty onset requires further research.

In addition, this study determined the levels of each type of mC base. We found that in the promoter regions, the methylation level was distributed more evenly; however, high methylation levels (> 90%) were more apparent in the CGI regions (about 25%). In a 2008 study, Cokus et al. found that methylation levels polarize; if an mC is not in a low methylation state, it is in a high methylation state [46]. We consider such a difference is mainly due to different species; our study used rats, and different species have different characteristics of DNA methylation.

The effects of DNA methylation on the onset of puberty have been reported in previous studies. In particular, the hypothalamic expression of two key Polycomb group (PcG) genes, *Eed* and *Cbx7*, decreased, and methylation of their promoters increased before puberty [32]. Before the onset of puberty, Eed represses of *Kiss1* expression (which encodes the protein kisspeptin) by binding to the *Kiss1* promoter. Epigenetic silencing of Eed leads to its dissociation from the *Kiss1* promoter, resulting in an increase in *Kiss1* mRNA expression at the puberty onset. Indeed, the typicap increase in pulsatile GnRH release that occurs at puberty onset was disrupted when Eed remained bound to the *Kiss1* promoter, perhaps due to inhibition of DNA methylation [32]. Unfortunately, we did not find methylation changes in these two genes in our study. However, undetected promoter methylation changes may have occurred due to the absence of restriction enzyme sites used in the RRBS technology.

Pervious studies have also shown an abundance of H3K4me3 and H3K9ac14 at the Kiss1 promoter in prepuberty is associated with the loss of PcG inhibition [32, 47]. In particular, the TrxG (Trithorax group) complex, which antagonizes PcG proteins, catalyzes H3K4me3 and facilitates H3 acetylation. Some evidence indicates the TrxG complex is involved in the control of puberty, for example, CHD7 mutations result in hypothalamic hypogonadism in humans [48]. In particular, the TrxG complex may activate puberty-associated genes by post-translationally modifying histones during the onset of puberty [47]. However, we found no link between the TrxG complex and DNA methylation in our study. The involvement of post-translational modification of histones in the control of puberty onset is a future direction of our research.

We applied GO and KEGG pathway analysis of the DMRs in prepuberty and puberty. We found the vasopressin-regulated water reabsorption pathway was the most enriched in these pathways. Vasopressin is released due to high salt could,and could activate vasopressin receptors expressed on neurons that release kisspeptin in the anteroventral periventricular nucleus. This could stimulated GnRH and LH secretion, which affect puberty onset [49–53]. Regardless of whether the methylated regions were in the promoter or CGI, they are related to the GnRH signaling pathway (*Gnas* and *Itpr2*), and the key role of GnRH in puberty is well established [54]. In CGI, the methylated regions were linked to the oocyte meiosis signaling pathway (*Itpr2*).Although the methylation of *Gnas* and *Itpr2* were different, the expression level showed no significance from prepuberty to puberty, indicating the correclation between methylation levels yaries, which is consistent with previous studies [55–57]. Indeed, the role of *Gnas* and *Itpr2* in the onset of puberty requires further study.

Except for *Gnas* and *Itpr2*, we found that more than one differentially methylated gene was involved in a wide range of signaling pathways, including cellular processes, biological processes, biological regulation, cells and cell components; these are all closely related to growth and development. These results also indicate that the onset of puberty involves multiple signaling pathways simultaneously, and these signaling pathways could have crosstalk interactions. This further confirms the complexity and multifaceted nature of the regulatory mechanisms of puberty. Exactly which gene silencing or activation pathway plays a regulatory role in puberty onset, and their regulatory mechanisms, requires further screening and verification.

The puberty onset of the male is different from the female, and only female rats were examined in the manuscript. The differences between males and females will be solved in a future study. In addition, there was DNA methylation change in prepubertal and pubertal female samples. However, our data is not sufficient to prove that DNA methylation regulation onset of puberty, because so many factors can influence the epigenetic change, such as estrogen [58]. We will be exploring what specific factors cause this methylation changes and whether this changes can be used as a marker to indicate the puberty onset in the future research.

## Conclusions

Our results demonstrate changes in DNA methylation occur from prepubertal to puberty in rats, suggesting that DNA methylation may play a crucial role in the regulation of puberty onset. The study provides essential information for future studies of the role of epigenetics in puberty.

## Additional file

Additional file 1:
**Table** **S1.** Comparison of reads within reference sequence. **Table S2.** The number of promoters and CGI covered by RRBS. **Table S3.** The cellulara component term with differentially methylated genes in promoter. **Table S4.** The molecular function term with differentially methylated genes in promoter. **Table S5.** The biological process term with differentially methylated genes in promoter. **Table S6.** The cellulara component term with differentially methylated genes in CGI. **Table S7.** The molecular function term with differentially methylated genes inCGI. **Table S8.** The biological process term with differentially methylated genes in CGI. **Table S9.** The pathways with differentially methylated genes in promoter. **Table S10.** The pathways with differentially methylated genes in CGI. (DOC 830 kb)

## Abbreviations

CGI: Cytosine-phosphate-guanine island
CpG: Cytosine-phosphate-guanine
DMRs: Differentially methylated regions
DNA: Deoxynucleic acid
GnRH: Gonadotropin-releasing hormone
GO: Gene ontology
KEGG: Kyoto encyclopedia of genes and genomes
Kiss-1/GPR54: Gene encoding kisspeptin/G-protein receptor
NKB: Neurokinin B
NPY: Neuropeptide Y
RRBS: Reduced Representation Bisulfite Sequencing
