# Supplementary material for: Identification of differential genomic DNA Methylation in the hypothalamus of pubertal rat using reduced representation Bisulfite sequencing
Source: Reprod Biol Endocrinol. 2017 Oct 6;15:81. doi: 10.1186/s12958-017-0301-2 (PMC5639587; doi:10.1186/s12958-017-0301-2)
Supplement: Additional file 1: — Table S1. Comparison of reads within reference sequence. Table S2. The number of promoters and CGI covered by RRBS. Table S3. The cellulara component term with differentially methylated genes in promoter. Table S4. The molecular function term with differentially methylated genes in promoter. Table S5. The biological process term with differentially methylated genes in promoter. Table S6. The cellulara component term with differentially methylated genes in CGI. Table S7. The molecular function term with differentially methylated genes inCGI. Table S8. The biological process term with differentially methylated genes in CGI. Table S9. The pathways with differentially methylated genes in promoter. Table S10. The pathways with differentially methylated genes in CGI. (DOC 830 kb) [file 12958_2017_301_MOESM1_ESM.doc]

**Table S1. Comparison of reads within reference sequence**

| Sample | Prepuberty | Puberty |
| --- | --- | --- |
| Clean reads (M) | 248,727,316 | 244,131,868 |
| Mapped reads(M) | 240,319,388 | 236,299,433 |
| Map rate (%) | 96.62 | 96.79 |
| Uniquely mapped reads (M) | 194,708,481 | 187,361,492 |
| Uniquely mapped rate (%) | 78.28 | 76.75 |
| Enzyme rate (%) | 98.61 | 98.56 |
| Uniquely enzyme mapped rate (%) | 77.19 | 75.64 |
| Bisulfite conversion rate (%) | 99.32 | 99.39 |

**Table S2. The number of promoters and CGI covered by RRBS**

|  | | Promoter | | | CGI | | |
| --- | --- | --- | --- | --- | --- | --- | --- |
|  |  | CG | CHG | CHH | CG | CHG | CHH |
| Genome | | 13,714 | 34,767 | 107,908 | 2,605,360 | 2,108,472 | 4,111,032 |
| Target region | | 13,714 | 34,769 | 107,924 | 2,605,322 | 2,108,514 | 4,111,734 |
| Prepuberty | Number | 4,894 | 6,472 | 14,635 | 1,359,226 | 1,133,535 | 2,062,809 |
|  | Rate (%) | 35.69 | 18.61 | 13.56 | 52.17 | 53.76 | 50.17 |
| Puberty | Number | 4,616 | 5,898 | 13,106 | 1,338,206 | 1,115,821 | 2,027,863 |
|  | Rate (%) | 33.66 | 16.96 | 12.14 | 51.36 | 52.92 | 49.32 |

**Table S3.The cellulara component term with differentially methylated genes in promoter**

| **Gene Ontology term** | **Cluster frequency** | **Genes annotated to the term** | **Corrected P-value** |
| --- | --- | --- | --- |
| organelle membrane | 2 out of 6 genes, 33.3% | 83533, 24896 | 1 |
| Intracellular membrane-bounded organelle | 5 out of 6 genes, 83.3% | 314746, 83533, 29361, 24896, 25177 | 1 |
| membrane-bounded organelle | 5 out of 6 genes, 83.3% | 314746, 83533, 29361, 24896, 25177 | 1 |
| intracellular organelle | 5 out of 6 genes, 83.3% | 314746, 83533, 29361, 24896, 25177 | 1 |
| organelle | 5 out of 6 genes, 83.3% | 314746, 83533, 29361, 24896, 25177 | 1 |
| cytoplasmic part | 3 out of 6 genes, 50.0% | 83533, 24896, 25177 | 1 |
| cytoplasm | 3 out of 6 genes, 50.0% | 83533, 24896, 25177 | 1 |
| intracellular part | 5 out of 6 genes, 83.3% | 314746, 83533, 29361, 24896, 25177 | 1 |
| intracellular | 5 out of 6 genes, 83.3% | 314746, 83533, 29361, 24896, 25177 | 1 |
| intracellular organelle part | 2 out of 6 genes, 33.3% | 83533, 24896 | 1 |
| membrane | 3 out of 6 genes, 50.0% | 83533, 24896, 25177 | 1 |
| cell | 5 out of 6 genes, 83.3% | 314746, 83533, 29361, 24896, 25177 | 1 |
| cell part | 5 out of 6 genes, 83.3% | 314746, 83533, 29361, 24896, 25177 | 1 |
| organelle part | 2 out of 6 genes, 33.3% | 83533, 24896 | 1 |

**Table S4.The molecular function term with differentially methylated genes in promoter**

| **Gene Ontology term** | **Cluster frequency** | **Genes annotated to the term** | **Corrected P-value** |
| --- | --- | --- | --- |
| G-protein coupled receptor binding | 2 out of 6 genes, 33.3% | 366270, 24896 | 0.033 |
| receptor binding | 2 out of 6 genes, 33.3% | 366270, 24896 | 1 |
| transition metal ion binding | 2 out of 6 genes, 33.3% | 314746, 25177 | 1 |
| binding | 6 out of 6 genes,100.0% | 314746, 366270, 83533, 29361, 24896, 25177 | 1 |
| heterocyclic compound binding | 3 out of 6 genes, 50.0% | 83533, 29361, 24896 | 1 |
| organic cyclic compound binding | 3 out of 6 genes, 50.0% | 83533, 29361, 24896 | 1 |
| hydrolase activity | 2 out of 6 genes, 33.3% | 314746, 24896 | 1 |
| nucleic acid binding | 2 out of 6 genes, 33.3% | 83533, 29361 | 1 |
| protein binding | 3 out of 6 genes, 50.0% | 366270, 29361, 24896 | 1 |
| metal ion binding | 2 out of 6 genes, 33.3% | 314746, 25177 | 1 |
| catalytic activity | 3 out of 6 genes, 50.0% | 314746, 83533, 24896 | 1 |
| cation binding | 2 out of 6 genes, 33.3% | 314746, 25177 | 1 |
| ion binding | 2 out of 6 genes, 33.3% | 314746, 25177 | 1 |

**Table S5.The biological process term with differentially methylated genes in promoter**

| **Gene Ontology term** | **Cluster frequency** | **Genes annotated to the term** | **Corrected P-value** |
| --- | --- | --- | --- |
| [pigment cell differentiation](http://amigo.geneontology.org/cgi-bin/amigo/go.cgi?action=query&view=query&query=GO:0050931&search_constraint=terms) | 2 out of 7 genes, 28.6% | 366270, 29361 | 0.00523 |
| [developmental pigmentation](http://amigo.geneontology.org/cgi-bin/amigo/go.cgi?action=query&view=query&query=GO:0048066&search_constraint=terms) | 2 out of 7 genes, 28.6% | 366270, 29361 | 0.00657 |
| [pigmentation](http://amigo.geneontology.org/cgi-bin/amigo/go.cgi?action=query&view=query&query=GO:0043473&search_constraint=terms) | 2 out of 7 genes, 28.6% | 366270, 29361 | 0.02274 |
| [peptide secretion](http://amigo.geneontology.org/cgi-bin/amigo/go.cgi?action=query&view=query&query=GO:0002790&search_constraint=terms) | 2 out of 7 genes, 28.6% | 366270, 24896 | 0.16356 |
| [growth](http://amigo.geneontology.org/cgi-bin/amigo/go.cgi?action=query&view=query&query=GO:0040007&search_constraint=terms) | 3 out of 7 genes, 42.9% | 29361, 24896, 25177 | 0.20547 |
| [peptide transport](http://amigo.geneontology.org/cgi-bin/amigo/go.cgi?action=query&view=query&query=GO:0015833&search_constraint=terms) | 2 out of 7 genes, 28.6% | 366270, 24896 | 0.20734 |
| [amide transport](http://amigo.geneontology.org/cgi-bin/amigo/go.cgi?action=query&view=query&query=GO:0042886&search_constraint=terms) | 2 out of 7 genes, 28.6% | 366270, 24896 | 0.23109 |
| [single-organism cellular process](http://amigo.geneontology.org/cgi-bin/amigo/go.cgi?action=query&view=query&query=GO:0044763&search_constraint=terms) | 7 out of 7 genes, 100.0% | 314746, 366270, 83533, 299266, 29361, 24896, 25177 | 1 |
| [chordate embryonic development](http://amigo.geneontology.org/cgi-bin/amigo/go.cgi?action=query&view=query&query=GO:0043009&search_constraint=terms) | 2 out of 7 genes, 28.6% | 29361, 24896 | 1 |
| [embryo development ending in birth or egg hatching](http://amigo.geneontology.org/cgi-bin/amigo/go.cgi?action=query&view=query&query=GO:0009792&search_constraint=terms) | 2 out of 7 genes, 28.6% | 29361, 24896 | 1 |
| [secretion](http://amigo.geneontology.org/cgi-bin/amigo/go.cgi?action=query&view=query&query=GO:0046903&search_constraint=terms) | 2 out of 7 genes, 28.6% | 366270, 24896 | 1 |
| [nitrogen compound transport](http://amigo.geneontology.org/cgi-bin/amigo/go.cgi?action=query&view=query&query=GO:0071705&search_constraint=terms) | 2 out of 7 genes, 28.6% | 366270, 24896 | 1 |
| [anatomical structure morphogenesis](http://amigo.geneontology.org/cgi-bin/amigo/go.cgi?action=query&view=query&query=GO:0009653&search_constraint=terms) | 3 out of 7 genes, 42.9% | 29361, 24896, 25177 | 1 |
| [DNA metabolic process](http://amigo.geneontology.org/cgi-bin/amigo/go.cgi?action=query&view=query&query=GO:0006259&search_constraint=terms) | 2 out of 7 genes, 28.6% | 83533, 24896 | 1 |
| [regulation of cellular protein metabolic process](http://amigo.geneontology.org/cgi-bin/amigo/go.cgi?action=query&view=query&query=GO:0032268&search_constraint=terms) | 2 out of 7 genes, 28.6% | 314746, 366270 | 1 |
| [regulation of immune system process](http://amigo.geneontology.org/cgi-bin/amigo/go.cgi?action=query&view=query&query=GO:0002682&search_constraint=terms) | 2 out of 7 genes, 28.6% | 366270, 24896 | 1 |
| [nucleobase-containing compound catabolic process](http://amigo.geneontology.org/cgi-bin/amigo/go.cgi?action=query&view=query&query=GO:0034655&search_constraint=terms) | 2 out of 7 genes, 28.6% | 83533, 24896 | 1 |
| [aromatic compound catabolic process](http://amigo.geneontology.org/cgi-bin/amigo/go.cgi?action=query&view=query&query=GO:0019439&search_constraint=terms) | 2 out of 7 genes, 28.6% | 83533, 24896 | 1 |
| [heterocycle catabolic process](http://amigo.geneontology.org/cgi-bin/amigo/go.cgi?action=query&view=query&query=GO:0046700&search_constraint=terms) | 2 out of 7 genes, 28.6% | 83533, 24896 | 1 |
| [cellular nitrogen compound catabolic process](http://amigo.geneontology.org/cgi-bin/amigo/go.cgi?action=query&view=query&query=GO:0044270&search_constraint=terms) | 2 out of 7 genes, 28.6% | 83533, 24896 | 1 |
| [single-organism process](http://amigo.geneontology.org/cgi-bin/amigo/go.cgi?action=query&view=query&query=GO:0044699&search_constraint=terms) | 7 out of 7 genes, 100.0% | 314746, 366270, 83533, 299266, 29361, 24896, 25177 | 1 |
| [organic cyclic compound catabolic process](http://amigo.geneontology.org/cgi-bin/amigo/go.cgi?action=query&view=query&query=GO:1901361&search_constraint=terms) | 2 out of 7 genes, 28.6% | 83533, 24896 | 1 |
| [embryo development](http://amigo.geneontology.org/cgi-bin/amigo/go.cgi?action=query&view=query&query=GO:0009790&search_constraint=terms) | 2 out of 7 genes, 28.6% | 29361, 24896 | 1 |
| [regulation of protein metabolic process](http://amigo.geneontology.org/cgi-bin/amigo/go.cgi?action=query&view=query&query=GO:0051246&search_constraint=terms) | 2 out of 7 genes, 28.6% | 314746, 366270 | 1 |
| [biological regulation](http://amigo.geneontology.org/cgi-bin/amigo/go.cgi?action=query&view=query&query=GO:0065007&search_constraint=terms) | 6 out of 7 genes, 85.7% | 314746, 366270, 83533, 299266, 29361, 24896 | 1 |
| [cellular developmental process](http://amigo.geneontology.org/cgi-bin/amigo/go.cgi?action=query&view=query&query=GO:0048869&search_constraint=terms) | 3 out of 7 genes, 42.9% | 366270, 29361, 25177 | 1 |
| [regulation of developmental process](http://amigo.geneontology.org/cgi-bin/amigo/go.cgi?action=query&view=query&query=GO:0050793&search_constraint=terms) | 2 out of 7 genes, 28.6% | 366270, 24896 | 1 |
| [regulation of biological quality](http://amigo.geneontology.org/cgi-bin/amigo/go.cgi?action=query&view=query&query=GO:0065008&search_constraint=terms) | 3 out of 7 genes, 42.9% | 366270, 83533, 24896 | 1 |
| [regulation of macromolecule metabolic process](http://amigo.geneontology.org/cgi-bin/amigo/go.cgi?action=query&view=query&query=GO:0060255&search_constraint=terms) | 3 out of 7 genes, 42.9% | 314746, 366270, 29361 | 1 |
| [cell proliferation](http://amigo.geneontology.org/cgi-bin/amigo/go.cgi?action=query&view=query&query=GO:0008283&search_constraint=terms) | 2 out of 7 genes, 28.6% | 366270, 29361 | 1 |
| [regulation of primary metabolic process](http://amigo.geneontology.org/cgi-bin/amigo/go.cgi?action=query&view=query&query=GO:0080090&search_constraint=terms) | 3 out of 7 genes, 42.9% | 314746, 366270, 29361 | 1 |
| [developmental process](http://amigo.geneontology.org/cgi-bin/amigo/go.cgi?action=query&view=query&query=GO:0032502&search_constraint=terms) | 4 out of 7 genes, 57.1% | 366270, 29361, 24896, 25177 | 1 |
| [regulation of cellular metabolic process](http://amigo.geneontology.org/cgi-bin/amigo/go.cgi?action=query&view=query&query=GO:0031323&search_constraint=terms) | 3 out of 7 genes, 42.9% | 314746, 366270, 29361 | 1 |
| [homeostatic process](http://amigo.geneontology.org/cgi-bin/amigo/go.cgi?action=query&view=query&query=GO:0042592&search_constraint=terms) | 2 out of 7 genes, 28.6% | 83533, 24896 | 1 |
| [regulation of cellular process](http://amigo.geneontology.org/cgi-bin/amigo/go.cgi?action=query&view=query&query=GO:0050794&search_constraint=terms) | 5 out of 7 genes, 71.4% | 314746, 366270, 299266, 29361, 24896 | 1 |
| [cellular catabolic process](http://amigo.geneontology.org/cgi-bin/amigo/go.cgi?action=query&view=query&query=GO:0044248&search_constraint=terms) | 2 out of 7 genes, 28.6% | 83533, 24896 | 1 |
| [regulation of multicellular organismal process](http://amigo.geneontology.org/cgi-bin/amigo/go.cgi?action=query&view=query&query=GO:0051239&search_constraint=terms) | 2 out of 7 genes, 28.6% | 366270, 24896 | 1 |
| [organic substance catabolic process](http://amigo.geneontology.org/cgi-bin/amigo/go.cgi?action=query&view=query&query=GO:1901575&search_constraint=terms) | 2 out of 7 genes, 28.6% | 83533, 24896 | 1 |
| [organic substance transport](http://amigo.geneontology.org/cgi-bin/amigo/go.cgi?action=query&view=query&query=GO:0071702&search_constraint=terms) | 2 out of 7 genes, 28.6% | 366270, 24896 | 1 |
| [catabolic process](http://amigo.geneontology.org/cgi-bin/amigo/go.cgi?action=query&view=query&query=GO:0009056&search_constraint=terms) | 2 out of 7 genes, 28.6% | 83533, 24896 | 1 |
| [cellular process](http://amigo.geneontology.org/cgi-bin/amigo/go.cgi?action=query&view=query&query=GO:0009987&search_constraint=terms) | 7 out of 7 genes, 100.0% | 314746, 366270, 83533, 299266, 29361, 24896, 25177 | 1 |
| [single-multicellular organism process](http://amigo.geneontology.org/cgi-bin/amigo/go.cgi?action=query&view=query&query=GO:0044707&search_constraint=terms) | 4 out of 7 genes, 57.1% | 366270, 29361, 24896, 25177 | 1 |
| [system development](http://amigo.geneontology.org/cgi-bin/amigo/go.cgi?action=query&view=query&query=GO:0048731&search_constraint=terms) | 3 out of 7 genes, 42.9% | 29361, 24896, 25177 | 1 |
| [regulation of biological process](http://amigo.geneontology.org/cgi-bin/amigo/go.cgi?action=query&view=query&query=GO:0050789&search_constraint=terms) | 5 out of 7 genes, 71.4% | 314746, 366270, 299266, 29361, 24896 | 1 |
| [regulation of metabolic process](http://amigo.geneontology.org/cgi-bin/amigo/go.cgi?action=query&view=query&query=GO:0019222&search_constraint=terms) | 3 out of 7 genes, 42.9% | 314746, 366270, 29361 | 1 |
| [multicellular organismal development](http://amigo.geneontology.org/cgi-bin/amigo/go.cgi?action=query&view=query&query=GO:0007275&search_constraint=terms) | 3 out of 7 genes, 42.9% | 29361, 24896, 25177 | 1 |
| [positive regulation of cellular process](http://amigo.geneontology.org/cgi-bin/amigo/go.cgi?action=query&view=query&query=GO:0048522&search_constraint=terms) | 2 out of 7 genes, 28.6% | 366270, 24896 | 1 |
| [system process](http://amigo.geneontology.org/cgi-bin/amigo/go.cgi?action=query&view=query&query=GO:0003008&search_constraint=terms) | 2 out of 7 genes, 28.6% | 366270, 24896 | 1 |
| [cell differentiation](http://amigo.geneontology.org/cgi-bin/amigo/go.cgi?action=query&view=query&query=GO:0030154&search_constraint=terms) | 2 out of 7 genes, 28.6% | 366270, 29361 | 1 |
| [multicellular organismal process](http://amigo.geneontology.org/cgi-bin/amigo/go.cgi?action=query&view=query&query=GO:0032501&search_constraint=terms) | 4 out of 7 genes, 57.1% | 366270, 29361, 24896, 25177 | 1 |
| [anatomical structure development](http://amigo.geneontology.org/cgi-bin/amigo/go.cgi?action=query&view=query&query=GO:0048856&search_constraint=terms) | 3 out of 7 genes, 42.9% | 29361, 24896, 25177 | 1 |
| [signal transduction](http://amigo.geneontology.org/cgi-bin/amigo/go.cgi?action=query&view=query&query=GO:0007165&search_constraint=terms) | 3 out of 7 genes, 42.9% | 366270, 299266, 24896 | 1 |
| [organ development](http://amigo.geneontology.org/cgi-bin/amigo/go.cgi?action=query&view=query&query=GO:0048513&search_constraint=terms) | 2 out of 7 genes, 28.6% | 24896, 25177 | 1 |
| [positive regulation of biological process](http://amigo.geneontology.org/cgi-bin/amigo/go.cgi?action=query&view=query&query=GO:0048518&search_constraint=terms) | 2 out of 7 genes, 28.6% | 366270, 24896 | 1 |
| [nucleic acid metabolic process](http://amigo.geneontology.org/cgi-bin/amigo/go.cgi?action=query&view=query&query=GO:0090304&search_constraint=terms) | 2 out of 7 genes, 28.6% | 83533, 24896 | 1 |
| [macromolecule modification](http://amigo.geneontology.org/cgi-bin/amigo/go.cgi?action=query&view=query&query=GO:0043412&search_constraint=terms) | 2 out of 7 genes, 28.6% | 314746, 24896 | 1 |
| [single-organism transport](http://amigo.geneontology.org/cgi-bin/amigo/go.cgi?action=query&view=query&query=GO:0044765&search_constraint=terms) | 2 out of 7 genes, 28.6% | 366270, 24896 | 1 |
| [single organism signaling](http://amigo.geneontology.org/cgi-bin/amigo/go.cgi?action=query&view=query&query=GO:0044700&search_constraint=terms) | 3 out of 7 genes, 42.9% | 366270, 299266, 24896 | 1 |
| [cellular response to stimulus](http://amigo.geneontology.org/cgi-bin/amigo/go.cgi?action=query&view=query&query=GO:0051716&search_constraint=terms) | 3 out of 7 genes, 42.9% | 366270, 299266, 24896 | 1 |
| [cell communication](http://amigo.geneontology.org/cgi-bin/amigo/go.cgi?action=query&view=query&query=GO:0007154&search_constraint=terms) | 3 out of 7 genes, 42.9% | 366270, 299266, 24896 | 1 |
| [cellular macromolecule metabolic process](http://amigo.geneontology.org/cgi-bin/amigo/go.cgi?action=query&view=query&query=GO:0044260&search_constraint=terms) | 3 out of 7 genes, 42.9% | 314746, 83533, 24896 | 1 |
| [single-organism developmental process](http://amigo.geneontology.org/cgi-bin/amigo/go.cgi?action=query&view=query&query=GO:0044767&search_constraint=terms) | 2 out of 7 genes, 28.6% | 29361, 24896 | 1 |
| [nucleobase-containing compound metabolic process](http://amigo.geneontology.org/cgi-bin/amigo/go.cgi?action=query&view=query&query=GO:0006139&search_constraint=terms) | 2 out of 7 genes, 28.6% | 83533, 24896 | 1 |
| [response to chemical stimulus](http://amigo.geneontology.org/cgi-bin/amigo/go.cgi?action=query&view=query&query=GO:0042221&search_constraint=terms) | 2 out of 7 genes, 28.6% | 366270, 24896 | 1 |
| [heterocycle metabolic process](http://amigo.geneontology.org/cgi-bin/amigo/go.cgi?action=query&view=query&query=GO:0046483&search_constraint=terms) | 2 out of 7 genes, 28.6% | 83533, 24896 | 1 |
| [cellular aromatic compound metabolic process](http://amigo.geneontology.org/cgi-bin/amigo/go.cgi?action=query&view=query&query=GO:0006725&search_constraint=terms) | 2 out of 7 genes, 28.6% | 83533, 24896 | 1 |
| [cellular nitrogen compound metabolic process](http://amigo.geneontology.org/cgi-bin/amigo/go.cgi?action=query&view=query&query=GO:0034641&search_constraint=terms) | 2 out of 7 genes, 28.6% | 83533, 24896 | 1 |
| [macromolecule metabolic process](http://amigo.geneontology.org/cgi-bin/amigo/go.cgi?action=query&view=query&query=GO:0043170&search_constraint=terms) | 3 out of 7 genes, 42.9% | 314746, 83533, 24896 | 1 |
| [transport](http://amigo.geneontology.org/cgi-bin/amigo/go.cgi?action=query&view=query&query=GO:0006810&search_constraint=terms) | 2 out of 7 genes, 28.6% | 366270, 24896 | 1 |
| [organic cyclic compound metabolic process](http://amigo.geneontology.org/cgi-bin/amigo/go.cgi?action=query&view=query&query=GO:1901360&search_constraint=terms) | 2 out of 7 genes, 28.6% | 83533, 24896 | 1 |
| [nitrogen compound metabolic process](http://amigo.geneontology.org/cgi-bin/amigo/go.cgi?action=query&view=query&query=GO:0006807&search_constraint=terms) | 2 out of 7 genes, 28.6% | 83533, 24896 | 1 |
| [establishment of localization](http://amigo.geneontology.org/cgi-bin/amigo/go.cgi?action=query&view=query&query=GO:0051234&search_constraint=terms) | 2 out of 7 genes, 28.6% | 366270, 24896 | 1 |
| [signaling](http://amigo.geneontology.org/cgi-bin/amigo/go.cgi?action=query&view=query&query=GO:0023052&search_constraint=terms) | 3 out of 7 genes, 42.9% | 366270, 299266, 24896 | 1 |
| [localization](http://amigo.geneontology.org/cgi-bin/amigo/go.cgi?action=query&view=query&query=GO:0051179&search_constraint=terms) | 2 out of 7 genes, 28.6% | 366270, 24896 | 1 |
| [response to stimulus](http://amigo.geneontology.org/cgi-bin/amigo/go.cgi?action=query&view=query&query=GO:0050896&search_constraint=terms) | 3 out of 7 genes, 42.9% | 366270, 299266, 24896 | 1 |
| [primary metabolic process](http://amigo.geneontology.org/cgi-bin/amigo/go.cgi?action=query&view=query&query=GO:0044238&search_constraint=terms) | 3 out of 7 genes, 42.9% | 314746, 83533, 24896 | 1 |
| [cellular metabolic process](http://amigo.geneontology.org/cgi-bin/amigo/go.cgi?action=query&view=query&query=GO:0044237&search_constraint=terms) | 3 out of 7 genes, 42.9% | 314746, 83533, 24896 | 1 |
| [organic substance metabolic process](http://amigo.geneontology.org/cgi-bin/amigo/go.cgi?action=query&view=query&query=GO:0071704&search_constraint=terms) | 3 out of 7 genes, 42.9% | 314746, 83533, 24896 | 1 |
| [metabolic process](http://amigo.geneontology.org/cgi-bin/amigo/go.cgi?action=query&view=query&query=GO:0008152&search_constraint=terms) | 3 out of 7 genes, 42.9% | 314746, 83533, 24896 | 1 |

**Table S6.The cellulara component term with differentially methylated genes in** CGI

| **Gene Ontology term** | **Cluster frequency** | **Genes annotated to the term** | **Corrected P-value** |
| --- | --- | --- | --- |
| cytoplasmic membrane-bounded vesicle | 5 out of 31 genes, 16.1% | 81678, 24944, 29714, 287709, 24896 | 1 |
| endomembrane system | 6 out of 31 genes, 19.4% | 81678, 299907, 24944, 64445, 64553, 24896 | 1 |
| membrane-bounded vesicle | 5 out of 31 genes, 16.1% | 81678, 24944, 29714, 287709, 24896 | 1 |
| Golgi membrane | 2 out of 31 genes, 6.5% | 64445, 24896 | 1 |
| cytoplasmic vesicle | 5 out of 31 genes, 16.1% | 81678, 24944, 29714, 287709, 24896 | 1 |
| endosome | 3 out of 31 genes, 9.7% | 297096, 24944, 287709 | 1 |
| endoplasmic reticulum | 3 out of 31 genes, 9.7% | 81678, 299907, 64553 | 1 |
| endoplasmic reticulum membrane | 2 out of 31 genes, 6.5% | 81678, 299907 | 1 |
| nuclear outer membrane-endoplasmic reticulum membrane network | 2 out of 31 genes, 6.5% | 81678, 299907 | 1 |
| intrinsic to organelle membrane | 2 out of 31 genes, 6.5% | 299907, 64445 | 1 |
| vesicle | 5 out of 31 genes, 16.1% | 81678, 24944, 29714, 287709, 24896 | 1 |
| organelle membrane | 7 out of 31 genes, 22.6% | 81678, 299907, 297096, 24944, 64445, 64553, 24896 | 1 |
| membrane | 19 out of 31 genes, 61.3% | 64445, 64553, 362945, 24896, 25177, 81678, 306141, 287709, 29714, 24944, 79219, 297604, 299907, 300653, 297096, 308022, 29185, 280670, 94268 | 1 |
| endoplasmic reticulum part | 2 out of 31 genes, 6.5% | 81678, 299907 | 1 |
| neuron projection | 3 out of 31 genes, 9.7% | 81678, 24413, 29714 | 1 |
| cytoplasmic part | 13 out of 31 genes, 41.9% | 24413, 24944, 29714, 369017, 64445, 64553, 24896, 25177, 299907, 81678, 297096, 306141, 287709 | 1 |
| extracellular matrix | 2 out of 31 genes, 6.5% | 300653, 85251 | 1 |
| cytoplasm | 13 out of 31 genes, 41.9% | 24413, 24944, 29714, 369017, 64445, 64553, 24896, 25177, 299907, 81678, 297096, 306141, 287709 | 1 |
| neuron part | 3 out of 31 genes, 9.7% | 81678, 24413, 29714 | 1 |
| Golgi apparatus part | 2 out of 31 genes, 6.5% | 64445, 24896 | 1 |
| Golgi apparatus | 2 out of 31 genes, 6.5% | 64445, 24896 | 1 |
| cell projection | 4 out of 31 genes, 12.9% | 81678, 24413, 29714, 306141 | 1 |
| cytoskeleton | 4 out of 31 genes, 12.9% | 24413, 306141, 280670, 369017 | 1 |
| intrinsic to membrane | 12 out of 31 genes, 38.7% | 24944, 29714, 297604, 79219, 64445, 64553, 300653, 299907, 81678, 308022, 29185, 94268 | 1 |
| membrane part | 13 out of 31 genes, 41.9% | 24944, 29714, 297604, 79219, 64445, 64553, 24896, 300653, 299907, 81678, 308022, 29185, 94268 | 1 |
| cytoskeletal part | 2 out of 31 genes, 6.5% | 24413, 369017 | 1 |
| intracellular part | 23 out of 31 genes, 74.2% | 363256, 303678, 64445, 362456, 64553, 24896, 25177, 81678, 301056, 306141, 315059, 287709, 24413, 24944, 29714, 369017, 299907, 297096, 308022, 29589, 280670, 293976, 360973 | 1 |
| intracellular | 23 out of 31 genes, 74.2% | 363256, 303678, 64445, 362456, 64553, 24896, 25177, 81678, 301056, 306141, 315059, 287709, 24413, 24944, 29714, 369017, 299907, 297096, 308022, 29589, 280670, 293976, 360973 | 1 |
| cell | 25 out of 31 genes, 80.6% | 363256, 303678, 64445, 362456, 64553, 24896, 25177, 81678, 301056, 306141, 315059, 303901, 287709, 24413, 24944, 29714, 79219, 369017, 299907, 297096, 308022, 29589, 280670, 293976, 360973 | 1 |
| cell part | 25 out of 31 genes, 80.6% | 363256, 303678, 64445, 362456, 64553, 24896, 25177, 81678, 301056, 306141, 315059, 303901, 287709, 24413, 24944, 29714, 79219, 369017, 299907, 297096, 308022, 29589, 280670, 293976, 360973 | 1 |
| envelope | 2 out of 31 genes, 6.5% | 79219, 64553 | 1 |
| intracellular organelle | 19 out of 31 genes, 61.3% | 363256, 64445, 64553, 24896, 25177, 81678, 301056, 306141, 315059, 287709, 24413, 29714, 24944, 369017, 299907, 297096, 29589, 280670, 293976 | 1 |
| intracellular membrane-bounded organelle | 17 out of 31 genes, 54.8% | 363256, 64445, 64553, 24896, 25177, 81678, 301056, 315059, 287709, 29714, 24944, 24413, 369017, 299907, 29589, 297096, 293976 | 1 |
| organelle | 19 out of 31 genes, 61.3% | 363256, 64445, 64553, 24896, 25177, 81678, 301056, 306141, 315059, 287709, 24413, 29714, 24944, 369017, 299907, 297096, 29589, 280670, 293976 | 1 |
| membrane-bounded organelle | 17 out of 31 genes, 54.8% | 363256, 64445, 64553, 24896, 25177, 81678, 301056, 315059, 287709, 29714, 24944, 24413, 369017, 299907, 29589, 297096, 293976 | 1 |
| intracellular organelle part | 9 out of 31 genes, 29.0% | 24413, 24944, 369017, 64445, 64553, 24896, 299907, 81678, 297096 | 1 |
| integral to membrane | 3 out of 31 genes, 9.7% | 29714, 64553, 94268 | 1 |
| non-membrane-bounded organelle | 4 out of 31 genes, 12.9% | 24413, 306141, 280670, 369017 | 1 |
| intracellular non-membrane-bounded organelle | 4 out of 31 genes, 12.9% | 24413, 306141, 280670, 369017 | 1 |
| plasma membrane part | 2 out of 31 genes, 6.5% | 79219, 24896 | 1 |
| plasma membrane | 2 out of 31 genes, 6.5% | 79219, 24896 | 1 |
| cell periphery | 2 out of 31 genes, 6.5% | 79219, 24896 | 1 |
| organelle part | 9 out of 31 genes, 29.0% | 24413, 24944, 369017, 64445, 64553, 24896, 299907, 81678, 297096 | 1 |
| protein complex | 2 out of 31 genes, 6.5% | 369017, 64553 | 1 |
| nuclear part | 2 out of 31 genes, 6.5% | 24413, 64553 | 1 |
| nucleus | 2 out of 31 genes, 6.5% | 24413, 64553 | 1 |
| macromolecular complex | 3 out of 31 genes, 9.7% | 24413, 369017, 64553 | 1 |

**Table S7.The molecular function term with differentially methylated genes in**CGI

| **Gene Ontology term** | **Cluster frequency** | **Genes annotated to the term** | **Corrected P-value** |
| --- | --- | --- | --- |
| [ligand-gated ion channel activity](http://amigo.geneontology.org/cgi-bin/amigo/go.cgi?action=query&view=query&query=GO:0015276&search_constraint=terms) | 2 out of 28 genes, 7.1% | 81678, 79219 | 1 |
| [ligand-gated channel activity](http://amigo.geneontology.org/cgi-bin/amigo/go.cgi?action=query&view=query&query=GO:0022834&search_constraint=terms) | 2 out of 28 genes, 7.1% | 81678, 79219 | 1 |
| [phosphoric ester hydrolase activity](http://amigo.geneontology.org/cgi-bin/amigo/go.cgi?action=query&view=query&query=GO:0042578&search_constraint=terms) | 3 out of 28 genes, 10.7% | 301056, 29714, 361576 | 1 |
| [protein dimerization activity](http://amigo.geneontology.org/cgi-bin/amigo/go.cgi?action=query&view=query&query=GO:0046983&search_constraint=terms) | 3 out of 28 genes, 10.7% | 299907, 24413, 313596 | 1 |
| [ion gated channel activity](http://amigo.geneontology.org/cgi-bin/amigo/go.cgi?action=query&view=query&query=GO:0022839&search_constraint=terms) | 2 out of 28 genes, 7.1% | 81678, 79219 | 1 |
| [phospholipid binding](http://amigo.geneontology.org/cgi-bin/amigo/go.cgi?action=query&view=query&query=GO:0005543&search_constraint=terms) | 2 out of 28 genes, 7.1% | 81678, 297096 | 1 |
| [gated channel activity](http://amigo.geneontology.org/cgi-bin/amigo/go.cgi?action=query&view=query&query=GO:0022836&search_constraint=terms) | 2 out of 28 genes, 7.1% | 81678, 79219 | 1 |
| [lipid binding](http://amigo.geneontology.org/cgi-bin/amigo/go.cgi?action=query&view=query&query=GO:0008289&search_constraint=terms) | 3 out of 28 genes, 10.7% | 81678, 297096, 24413 | 1 |
| [structure-specific DNA binding](http://amigo.geneontology.org/cgi-bin/amigo/go.cgi?action=query&view=query&query=GO:0043566&search_constraint=terms) | 2 out of 28 genes, 7.1% | 29589, 24413 | 1 |
| [transferase activity, transferring glycosyl groups](http://amigo.geneontology.org/cgi-bin/amigo/go.cgi?action=query&view=query&query=GO:0016757&search_constraint=terms) | 2 out of 28 genes, 7.1% | 299907, 64445 | 1 |
| [phosphatase activity](http://amigo.geneontology.org/cgi-bin/amigo/go.cgi?action=query&view=query&query=GO:0016791&search_constraint=terms) | 2 out of 28 genes, 7.1% | 301056, 29714 | 1 |
| [enzyme activator activity](http://amigo.geneontology.org/cgi-bin/amigo/go.cgi?action=query&view=query&query=GO:0008047&search_constraint=terms) | 2 out of 28 genes, 7.1% | 306141, 362456 | 1 |
| [GTPase regulator activity](http://amigo.geneontology.org/cgi-bin/amigo/go.cgi?action=query&view=query&query=GO:0030695&search_constraint=terms) | 2 out of 28 genes, 7.1% | 362456, 24896 | 1 |
| [nucleoside-triphosphatase regulator activity](http://amigo.geneontology.org/cgi-bin/amigo/go.cgi?action=query&view=query&query=GO:0060589&search_constraint=terms) | 2 out of 28 genes, 7.1% | 362456, 24896 | 1 |
| [identical protein binding](http://amigo.geneontology.org/cgi-bin/amigo/go.cgi?action=query&view=query&query=GO:0042802&search_constraint=terms) | 3 out of 28 genes, 10.7% | 299907, 24413, 313596 | 1 |
| [hydrolase activity, acting on ester bonds](http://amigo.geneontology.org/cgi-bin/amigo/go.cgi?action=query&view=query&query=GO:0016788&search_constraint=terms) | 3 out of 28 genes, 10.7% | 301056, 29714, 361576 | 1 |
| [ion channel activity](http://amigo.geneontology.org/cgi-bin/amigo/go.cgi?action=query&view=query&query=GO:0005216&search_constraint=terms) | 2 out of 28 genes, 7.1% | 81678, 79219 | 1 |
| [substrate-specific channel activity](http://amigo.geneontology.org/cgi-bin/amigo/go.cgi?action=query&view=query&query=GO:0022838&search_constraint=terms) | 2 out of 28 genes, 7.1% | 81678, 79219 | 1 |
| [guanyl nucleotide binding](http://amigo.geneontology.org/cgi-bin/amigo/go.cgi?action=query&view=query&query=GO:0019001&search_constraint=terms) | 2 out of 28 genes, 7.1% | 287709, 24896 | 1 |
| [guanyl ribonucleotide binding](http://amigo.geneontology.org/cgi-bin/amigo/go.cgi?action=query&view=query&query=GO:0032561&search_constraint=terms) | 2 out of 28 genes, 7.1% | 287709, 24896 | 1 |
| [channel activity](http://amigo.geneontology.org/cgi-bin/amigo/go.cgi?action=query&view=query&query=GO:0015267&search_constraint=terms) | 2 out of 28 genes, 7.1% | 81678, 79219 | 1 |
| [passive transmembrane transporter activity](http://amigo.geneontology.org/cgi-bin/amigo/go.cgi?action=query&view=query&query=GO:0022803&search_constraint=terms) | 2 out of 28 genes, 7.1% | 81678, 79219 | 1 |
| [ion transmembrane transporter activity](http://amigo.geneontology.org/cgi-bin/amigo/go.cgi?action=query&view=query&query=GO:0015075&search_constraint=terms) | 3 out of 28 genes, 10.7% | 81678, 308022, 79219 | 1 |
| [nucleic acid binding transcription factor activity](http://amigo.geneontology.org/cgi-bin/amigo/go.cgi?action=query&view=query&query=GO:0001071&search_constraint=terms) | 3 out of 28 genes, 10.7% | 29589, 24413, 293976 | 1 |
| [substrate-specific transmembrane transporter activity](http://amigo.geneontology.org/cgi-bin/amigo/go.cgi?action=query&view=query&query=GO:0022891&search_constraint=terms) | 3 out of 28 genes, 10.7% | 81678, 308022, 79219 | 1 |
| [binding](http://amigo.geneontology.org/cgi-bin/amigo/go.cgi?action=query&view=query&query=GO:0005488&search_constraint=terms) | 23 out of 28 genes, 82.1% | 313022,363256, 313596, 362456, 64553, 24896, 25177, 81678, 301056, 306141, 287709, 29714, 24413, 297604, 79219, 369017, 299907, 297096, 29589, 85251, 280670, 293976, 94268 | 1 |
| [enzyme regulator activity](http://amigo.geneontology.org/cgi-bin/amigo/go.cgi?action=query&view=query&query=GO:0030234&search_constraint=terms) | 3 out of 28 genes, 10.7% | 306141,362456,24896 | 1 |
| [transmembrane transporter activity](http://amigo.geneontology.org/cgi-bin/amigo/go.cgi?action=query&view=query&query=GO:0022857&search_constraint=terms) | 3 out of 28 genes, 10.7% | 81678, 308022, 79219 | 1 |
| [receptor binding](http://amigo.geneontology.org/cgi-bin/amigo/go.cgi?action=query&view=query&query=GO:0005102&search_constraint=terms) | 3 out of 28 genes, 10.7% | 24413, 94268, 24896 | 1 |
| [protein kinase activity](http://amigo.geneontology.org/cgi-bin/amigo/go.cgi?action=query&view=query&query=GO:0004672&search_constraint=terms) | 2 out of 28 genes, 7.1% | 313022, 363256 | 1 |
| [phosphotransferase activity, alcohol group as acceptor](http://amigo.geneontology.org/cgi-bin/amigo/go.cgi?action=query&view=query&query=GO:0016773&search_constraint=terms) | 2 out of 28 genes, 7.1% | 313022, 363256 | 1 |
| [molecular transducer activity](http://amigo.geneontology.org/cgi-bin/amigo/go.cgi?action=query&view=query&query=GO:0060089&search_constraint=terms) | 7 out of 28 genes, 25.0% | 313022, 24413, 29714, 297604, 79219, 303901, 24896 | 1 |
| [substrate-specific transporter activity](http://amigo.geneontology.org/cgi-bin/amigo/go.cgi?action=query&view=query&query=GO:0022892&search_constraint=terms) | 3 out of 28 genes, 10.7% | 81678, 308022, 79219 | 1 |
| [nucleoside-triphosphatase activity](http://amigo.geneontology.org/cgi-bin/amigo/go.cgi?action=query&view=query&query=GO:0017111&search_constraint=terms) | 2 out of 28 genes, 7.1% | 287709, 24896 | 1 |
| [transporter activity](http://amigo.geneontology.org/cgi-bin/amigo/go.cgi?action=query&view=query&query=GO:0005215&search_constraint=terms) | 3 out of 28 genes, 10.7% | 81678, 308022, 79219 | 1 |
| [signal transducer activity](http://amigo.geneontology.org/cgi-bin/amigo/go.cgi?action=query&view=query&query=GO:0004871&search_constraint=terms) | 6 out of 28 genes, 21.4% | 313022, 24413, 29714, 297604, 79219, 303901 | 1 |
| [kinase activity](http://amigo.geneontology.org/cgi-bin/amigo/go.cgi?action=query&view=query&query=GO:0016301&search_constraint=terms) | 2 out of 28 genes, 7.1% | 313022, 363256 | 1 |
| [pyrophosphatase activity](http://amigo.geneontology.org/cgi-bin/amigo/go.cgi?action=query&view=query&query=GO:0016462&search_constraint=terms) | 2 out of 28 genes, 7.1% | 287709, 24896 | 1 |
| [hydrolase activity](http://amigo.geneontology.org/cgi-bin/amigo/go.cgi?action=query&view=query&query=GO:0016787&search_constraint=terms) | 5 out of 28 genes, 17.9% | 301056, 29714, 361576, 287709, 24896 | 1 |
| [hydrolase activity, acting on acid anhydrides, in phosphorus-containing anhydrides](http://amigo.geneontology.org/cgi-bin/amigo/go.cgi?action=query&view=query&query=GO:0016818&search_constraint=terms) | 2 out of 28 genes, 7.1% | 287709, 24896 | 1 |
| [hydrolase activity, acting on acid anhydrides](http://amigo.geneontology.org/cgi-bin/amigo/go.cgi?action=query&view=query&query=GO:0016817&search_constraint=terms) | 2 out of 28 genes, 7.1% | 287709, 24896 | 1 |
| [DNA binding](http://amigo.geneontology.org/cgi-bin/amigo/go.cgi?action=query&view=query&query=GO:0003677&search_constraint=terms) | 2 out of 28 genes, 7.1% | 29589, 24413 | 1 |
| [ribonucleotide binding](http://amigo.geneontology.org/cgi-bin/amigo/go.cgi?action=query&view=query&query=GO:0032553&search_constraint=terms) | 4 out of 28 genes, 14.3% | 313022, 363256, 287709, 24896 | 1 |
| [purine ribonucleotide binding](http://amigo.geneontology.org/cgi-bin/amigo/go.cgi?action=query&view=query&query=GO:0032555&search_constraint=terms) | 4 out of 28 genes, 14.3% | 313022, 363256, 287709, 24896 | 1 |
| [purine nucleotide binding](http://amigo.geneontology.org/cgi-bin/amigo/go.cgi?action=query&view=query&query=GO:0017076&search_constraint=terms) | 4 out of 28 genes, 14.3% | 313022, 363256, 287709, 24896 | 1 |
| [heterocyclic compound binding](http://amigo.geneontology.org/cgi-bin/amigo/go.cgi?action=query&view=query&query=GO:1901363&search_constraint=terms) | 8 out of 28 genes, 28.6% | 313022, 29589, 363256, 24413, 313596, 293976, 287709, 24896 | 1 |
| [transferase activity](http://amigo.geneontology.org/cgi-bin/amigo/go.cgi?action=query&view=query&query=GO:0016740&search_constraint=terms) | 4 out of 28 genes, 14.3% | 299907, 313022, 363256, 64445 | 1 |
| [organic cyclic compound binding](http://amigo.geneontology.org/cgi-bin/amigo/go.cgi?action=query&view=query&query=GO:0097159&search_constraint=terms) | 8 out of 28 genes, 28.6% | 313022, 29589, 363256, 24413, 313596, 293976, 287709, 24896 | 1 |
| [nucleotide binding](http://amigo.geneontology.org/cgi-bin/amigo/go.cgi?action=query&view=query&query=GO:0000166&search_constraint=terms) | 4 out of 28 genes, 14.3% | 313022, 363256, 287709, 24896 | 1 |
| [nucleoside phosphate binding](http://amigo.geneontology.org/cgi-bin/amigo/go.cgi?action=query&view=query&query=GO:1901265&search_constraint=terms) | 4 out of 28 genes, 14.3% | 313022, 363256, 287709, 24896 | 1 |
| [transferase activity, transferring phosphorus-containing groups](http://amigo.geneontology.org/cgi-bin/amigo/go.cgi?action=query&view=query&query=GO:0016772&search_constraint=terms) | 2 out of 28 genes, 7.1% | 313022, 363256 | 1 |
| [small molecule binding](http://amigo.geneontology.org/cgi-bin/amigo/go.cgi?action=query&view=query&query=GO:0036094&search_constraint=terms) | 4 out of 28 genes, 14.3% | 313022, 363256, 287709, 24896 | 1 |
| [nucleic acid binding](http://amigo.geneontology.org/cgi-bin/amigo/go.cgi?action=query&view=query&query=GO:0003676&search_constraint=terms) | 4 out of 28 genes, 14.3% | 29589, 24413, 313596, 293976 | 1 |
| [receptor activity](http://amigo.geneontology.org/cgi-bin/amigo/go.cgi?action=query&view=query&query=GO:0004872&search_constraint=terms) | 3 out of 28 genes, 10.7% | 81678, 24413, 79219 | 1 |
| [catalytic activity](http://amigo.geneontology.org/cgi-bin/amigo/go.cgi?action=query&view=query&query=GO:0003824&search_constraint=terms) | 9 out of 28 genes, 32.1% | 313022, 363256, 29714, 64445, 24896, 299907, 301056, 361576, 287709 | 1 |
| [adenyl ribonucleotide binding](http://amigo.geneontology.org/cgi-bin/amigo/go.cgi?action=query&view=query&query=GO:0032559&search_constraint=terms) | 2 out of 28 genes, 7.1% | 313022, 363256 | 1 |
| [adenyl nucleotide binding](http://amigo.geneontology.org/cgi-bin/amigo/go.cgi?action=query&view=query&query=GO:0030554&search_constraint=terms) | 2 out of 28 genes, 7.1% | 313022, 363256 | 1 |
| [cation binding](http://amigo.geneontology.org/cgi-bin/amigo/go.cgi?action=query&view=query&query=GO:0043169&search_constraint=terms) | 5 out of 28 genes, 17.9% | 313022, 24413, 301056, 293976, 25177 | 1 |
| [protein binding](http://amigo.geneontology.org/cgi-bin/amigo/go.cgi?action=query&view=query&query=GO:0005515&search_constraint=terms) | 7 out of 28 genes, 25.0% | 299907, 24413, 313596, 280670, 64553, 94268, 24896 | 1 |
| [ion binding](http://amigo.geneontology.org/cgi-bin/amigo/go.cgi?action=query&view=query&query=GO:0043167&search_constraint=terms) | 5 out of 28 genes, 17.9% | 313022, 24413, 301056, 293976, 25177 | 1 |
| [transition metal ion binding](http://amigo.geneontology.org/cgi-bin/amigo/go.cgi?action=query&view=query&query=GO:0046914&search_constraint=terms) | 2 out of 28 genes, 7.1% | 24413, 25177 | 1 |
| [signaling receptor activity](http://amigo.geneontology.org/cgi-bin/amigo/go.cgi?action=query&view=query&query=GO:0038023&search_constraint=terms) | 2 out of 28 genes, 7.1% | 24413, 79219 | 1 |
| [metal ion binding](http://amigo.geneontology.org/cgi-bin/amigo/go.cgi?action=query&view=query&query=GO:0046872&search_constraint=terms) | 3 out of 28 genes, 10.7% | 313022, 24413, 25177 | 1 |

**Table S8.The biological process term with differentially methylated genes in CGI**

| **Gene Ontology term** | **Cluster frequency** | **Genes annotated to the term** | **Corrected P-value** |
| --- | --- | --- | --- |
| [sensory perception of mechanical stimulus](http://amigo.geneontology.org/cgi-bin/amigo/go.cgi?action=query&view=query&query=GO:0050954&search_constraint=terms) | 4 out of 28 genes, 14.3% | 300653, 308022, 29589, 306141 | 0.04006 |
| [cellular developmental process](http://amigo.geneontology.org/cgi-bin/amigo/go.cgi?action=query&view=query&query=GO:0048869&search_constraint=terms) | 11 out of 28 genes, 39.3% | 363256, 369017, 25177, 29589, 308022, 85251, 306141, 293976, 315059, 303901, 94268 | 0.45985 |
| [organ development](http://amigo.geneontology.org/cgi-bin/amigo/go.cgi?action=query&view=query&query=GO:0048513&search_constraint=terms) | 10 out of 28 genes, 35.7% | 363256, 24413, 297604, 24896, 25177, 299907, 308022, 29589, 306141, 94268 | 1 |
| [sensory perception](http://amigo.geneontology.org/cgi-bin/amigo/go.cgi?action=query&view=query&query=GO:0007600&search_constraint=terms) | 6 out of 28 genes, 21.4% | 300653, 308022, 29589, 85251, 306141, 24896 | 1 |
| [developmental process](http://amigo.geneontology.org/cgi-bin/amigo/go.cgi?action=query&view=query&query=GO:0032502&search_constraint=terms) | 15 out of 28 genes, 53.6% | 363256, 24413, 297604, 369017, 24896, 25177, 299907, 308022, 29589, 85251, 306141, 293976, 315059, 303901, 94268 | 1 |
| [insulin secretion](http://amigo.geneontology.org/cgi-bin/amigo/go.cgi?action=query&view=query&query=GO:0030073&search_constraint=terms) | 2 out of 28 genes, 7.1% | 81678, 24896 | 1 |
| [generation of a signal involved in cell-cell signaling](http://amigo.geneontology.org/cgi-bin/amigo/go.cgi?action=query&view=query&query=GO:0003001&search_constraint=terms) | 3 out of 28 genes, 10.7% | 81678, 24413, 24896 | 1 |
| [signal release](http://amigo.geneontology.org/cgi-bin/amigo/go.cgi?action=query&view=query&query=GO:0023061&search_constraint=terms) | 3 out of 28 genes, 10.7% | 81678, 24413, 24896 | 1 |
| [regulation of hydrolase activity](http://amigo.geneontology.org/cgi-bin/amigo/go.cgi?action=query&view=query&query=GO:0051336&search_constraint=terms) | 5 out of 28 genes, 17.9% | 81678, 29714, 306141, 362456, 64553 | 1 |
| [regulation of GTPase activity](http://amigo.geneontology.org/cgi-bin/amigo/go.cgi?action=query&view=query&query=GO:0043087&search_constraint=terms) | 3 out of 28 genes, 10.7% | 29714, 306141, 362456 | 1 |
| [regulation of GTP catabolic process](http://amigo.geneontology.org/cgi-bin/amigo/go.cgi?action=query&view=query&query=GO:0033124&search_constraint=terms) | 3 out of 28 genes, 10.7% | 29714, 306141, 362456 | 1 |
| [forebrain development](http://amigo.geneontology.org/cgi-bin/amigo/go.cgi?action=query&view=query&query=GO:0030900&search_constraint=terms) | 3 out of 28 genes, 10.7% | 299907, 29589, 24413 | 1 |
| [regulation of nucleoside metabolic process](http://amigo.geneontology.org/cgi-bin/amigo/go.cgi?action=query&view=query&query=GO:0009118&search_constraint=terms) | 3 out of 28 genes, 10.7% | 29714, 306141, 362456 | 1 |
| [system development](http://amigo.geneontology.org/cgi-bin/amigo/go.cgi?action=query&view=query&query=GO:0048731&search_constraint=terms) | 11 out of 28 genes, 39.3% | 363256, 24413, 297604, 24896, 25177, 299907, 308022, 29589, 306141, 303901, 94268 | 1 |
| [regulation of nucleotide catabolic process](http://amigo.geneontology.org/cgi-bin/amigo/go.cgi?action=query&view=query&query=GO:0030811&search_constraint=terms) | 3 out of 28 genes, 10.7% | 29714, 306141, 362456 | 1 |
| [regulation of purine nucleotide catabolic process](http://amigo.geneontology.org/cgi-bin/amigo/go.cgi?action=query&view=query&query=GO:0033121&search_constraint=terms) | 3 out of 28 genes, 10.7% | 29714, 306141, 362456 | 1 |
| [peptide hormone secretion](http://amigo.geneontology.org/cgi-bin/amigo/go.cgi?action=query&view=query&query=GO:0030072&search_constraint=terms) | 2 out of 28 genes, 7.1% | 81678, 24896 | 1 |
| [cellular response to growth factor stimulus](http://amigo.geneontology.org/cgi-bin/amigo/go.cgi?action=query&view=query&query=GO:0071363&search_constraint=terms) | 2 out of 28 genes, 7.1% | 24413, 306141 | 1 |
| [regulation of purine nucleotide metabolic process](http://amigo.geneontology.org/cgi-bin/amigo/go.cgi?action=query&view=query&query=GO:1900542&search_constraint=terms) | 3 out of 28 genes, 10.7% | 29714, 306141, 362456 | 1 |
| [peptide secretion](http://amigo.geneontology.org/cgi-bin/amigo/go.cgi?action=query&view=query&query=GO:0002790&search_constraint=terms) | 2 out of 28 genes, 7.1% | 81678, 24896 | 1 |
| [regulation of catalytic activity](http://amigo.geneontology.org/cgi-bin/amigo/go.cgi?action=query&view=query&query=GO:0050790&search_constraint=terms) | 6 out of 28 genes, 21.4% | 81678, 313022, 29714, 306141, 362456, 64553 | 1 |
| [ear development](http://amigo.geneontology.org/cgi-bin/amigo/go.cgi?action=query&view=query&query=GO:0043583&search_constraint=terms) | 2 out of 28 genes, 7.1% | 308022, 306141 | 1 |
| [anatomical structure development](http://amigo.geneontology.org/cgi-bin/amigo/go.cgi?action=query&view=query&query=GO:0048856&search_constraint=terms) | 12 out of 28 genes, 42.9% | 363256, 24413, 297604, 24896, 25177, 299907, 308022, 29589, 85251, 306141, 303901, 94268 | 1 |
| [regulation of nucleotide metabolic process](http://amigo.geneontology.org/cgi-bin/amigo/go.cgi?action=query&view=query&query=GO:0006140&search_constraint=terms) | 3 out of 28 genes, 10.7% | 29714, 306141, 362456 | 1 |
| [hormone secretion](http://amigo.geneontology.org/cgi-bin/amigo/go.cgi?action=query&view=query&query=GO:0046879&search_constraint=terms) | 2 out of 28 genes, 7.1% | 81678, 24896 | 1 |
| [neurological system process](http://amigo.geneontology.org/cgi-bin/amigo/go.cgi?action=query&view=query&query=GO:0050877&search_constraint=terms) | 6 out of 28 genes, 21.4% | 300653, 308022, 29589, 85251, 306141, 24896 | 1 |
| [anatomical structure morphogenesis](http://amigo.geneontology.org/cgi-bin/amigo/go.cgi?action=query&view=query&query=GO:0009653&search_constraint=terms) | 7 out of 28 genes, 25.0% | 299907, 85251, 24413, 306141, 94268, 24896, 25177 | 1 |
| [peptide transport](http://amigo.geneontology.org/cgi-bin/amigo/go.cgi?action=query&view=query&query=GO:0015833&search_constraint=terms) | 2 out of 28 genes, 7.1% | 81678, 24896 | 1 |
| [regulation of phosphate metabolic process](http://amigo.geneontology.org/cgi-bin/amigo/go.cgi?action=query&view=query&query=GO:0019220&search_constraint=terms) | 5 out of 28 genes, 17.9% | 313022, 29714, 306141, 362456, 64553 | 1 |
| [regulation of phosphorus metabolic process](http://amigo.geneontology.org/cgi-bin/amigo/go.cgi?action=query&view=query&query=GO:0051174&search_constraint=terms) | 5 out of 28 genes, 17.9% | 313022, 29714, 306141, 362456, 64553 | 1 |
| [multicellular organismal development](http://amigo.geneontology.org/cgi-bin/amigo/go.cgi?action=query&view=query&query=GO:0007275&search_constraint=terms) | 11 out of 28 genes, 39.3% | 363256, 24413, 297604, 24896, 25177, 299907, 308022, 29589, 306141, 303901, 94268 | 1 |
| [respiratory tube development](http://amigo.geneontology.org/cgi-bin/amigo/go.cgi?action=query&view=query&query=GO:0030323&search_constraint=terms) | 2 out of 28 genes, 7.1% | 24413, 306141 | 1 |
| [amide transport](http://amigo.geneontology.org/cgi-bin/amigo/go.cgi?action=query&view=query&query=GO:0042886&search_constraint=terms) | 2 out of 28 genes, 7.1% | 81678, 24896 | 1 |
| [positive regulation of MAPK cascade](http://amigo.geneontology.org/cgi-bin/amigo/go.cgi?action=query&view=query&query=GO:0043410&search_constraint=terms) | 2 out of 28 genes, 7.1% | 313022, 306141 | 1 |
| [single-multicellular organism process](http://amigo.geneontology.org/cgi-bin/amigo/go.cgi?action=query&view=query&query=GO:0044707&search_constraint=terms) | 14 out of 28 genes, 50.0% | 363256, 24413, 297604, 24896, 25177, 300653, 299907, 81678, 308022, 29589, 85251, 306141, 303901, 94268 | 1 |
| [regulation of MAP kinase activity](http://amigo.geneontology.org/cgi-bin/amigo/go.cgi?action=query&view=query&query=GO:0043405&search_constraint=terms) | 2 out of 28 genes, 7.1% | 313022, 306141 | 1 |
| [hormone transport](http://amigo.geneontology.org/cgi-bin/amigo/go.cgi?action=query&view=query&query=GO:0009914&search_constraint=terms) | 2 out of 28 genes, 7.1% | 81678, 24896 | 1 |
| [multicellular organismal process](http://amigo.geneontology.org/cgi-bin/amigo/go.cgi?action=query&view=query&query=GO:0032501&search_constraint=terms) | 15 out of 28 genes, 53.6% | 363256, 24413, 297604, 24896, 25177, 300653, 299907, 81678, 308022, 29589, 85251, 306141, 293976, 303901, 94268 | 1 |
| [system process](http://amigo.geneontology.org/cgi-bin/amigo/go.cgi?action=query&view=query&query=GO:0003008&search_constraint=terms) | 7 out of 28 genes, 25.0% | 300653, 308022, 29589, 85251, 24413, 306141, 24896 | 1 |
| [regulation of cellular catabolic process](http://amigo.geneontology.org/cgi-bin/amigo/go.cgi?action=query&view=query&query=GO:0031329&search_constraint=terms) | 3 out of 28 genes, 10.7% | 29714, 306141, 362456 | 1 |
| [secretion by cell](http://amigo.geneontology.org/cgi-bin/amigo/go.cgi?action=query&view=query&query=GO:0032940&search_constraint=terms) | 3 out of 28 genes, 10.7% | 81678, 24413, 24896 | 1 |
| [telencephalon development](http://amigo.geneontology.org/cgi-bin/amigo/go.cgi?action=query&view=query&query=GO:0021537&search_constraint=terms) | 2 out of 28 genes, 7.1% | 299907, 24413 | 1 |
| [brain development](http://amigo.geneontology.org/cgi-bin/amigo/go.cgi?action=query&view=query&query=GO:0007420&search_constraint=terms) | 3 out of 28 genes, 10.7% | 299907, 29589, 24413 | 1 |
| [single-organism developmental process](http://amigo.geneontology.org/cgi-bin/amigo/go.cgi?action=query&view=query&query=GO:0044767&search_constraint=terms) | 9 out of 28 genes, 32.1% | 24413, 297604, 24896, 299907, 29589, 308022, 85251, 306141, 94268 | 1 |
| [tissue development](http://amigo.geneontology.org/cgi-bin/amigo/go.cgi?action=query&view=query&query=GO:0009888&search_constraint=terms) | 5 out of 28 genes, 17.9% | 299907, 85251, 24413, 94268, 24896 | 1 |
| [response to growth factor stimulus](http://amigo.geneontology.org/cgi-bin/amigo/go.cgi?action=query&view=query&query=GO:0070848&search_constraint=terms) | 2 out of 28 genes, 7.1% | 24413, 306141 | 1 |
| [regulation of protein serine/threonine kinase activity](http://amigo.geneontology.org/cgi-bin/amigo/go.cgi?action=query&view=query&query=GO:0071900&search_constraint=terms) | 2 out of 28 genes, 7.1% | 313022, 306141 | 1 |
| [embryonic organ morphogenesis](http://amigo.geneontology.org/cgi-bin/amigo/go.cgi?action=query&view=query&query=GO:0048562&search_constraint=terms) | 2 out of 28 genes, 7.1% | 306141, 24896 | 1 |
| [regulation of intracellular protein kinase cascade](http://amigo.geneontology.org/cgi-bin/amigo/go.cgi?action=query&view=query&query=GO:0010627&search_constraint=terms) | 3 out of 28 genes, 10.7% | 313022, 306141, 64553 | 1 |
| [regulation of catabolic process](http://amigo.geneontology.org/cgi-bin/amigo/go.cgi?action=query&view=query&query=GO:0009894&search_constraint=terms) | 3 out of 28 genes, 10.7% | 29714, 306141, 362456 | 1 |
| [embryonic morphogenesis](http://amigo.geneontology.org/cgi-bin/amigo/go.cgi?action=query&view=query&query=GO:0048598&search_constraint=terms) | 3 out of 28 genes, 10.7% | 299907, 306141, 24896 | 1 |
| [cellular response to endogenous stimulus](http://amigo.geneontology.org/cgi-bin/amigo/go.cgi?action=query&view=query&query=GO:0071495&search_constraint=terms) | 2 out of 28 genes, 7.1% | 24413, 306141 | 1 |
| [regulation of molecular function](http://amigo.geneontology.org/cgi-bin/amigo/go.cgi?action=query&view=query&query=GO:0065009&search_constraint=terms) | 6 out of 28 genes, 21.4% | 81678, 313022, 29714, 306141, 362456, 64553 | 1 |
| [regulation of hormone levels](http://amigo.geneontology.org/cgi-bin/amigo/go.cgi?action=query&view=query&query=GO:0010817&search_constraint=terms) | 2 out of 28 genes, 7.1% | 81678, 24896 | 1 |
| [embryonic organ development](http://amigo.geneontology.org/cgi-bin/amigo/go.cgi?action=query&view=query&query=GO:0048568&search_constraint=terms) | 2 out of 28 genes, 7.1% | 306141, 24896 | 1 |
| [enzyme linked receptor protein signaling pathway](http://amigo.geneontology.org/cgi-bin/amigo/go.cgi?action=query&view=query&query=GO:0007167&search_constraint=terms) | 4 out of 28 genes, 14.3% | 81678, 24413, 306141, 94268 | 1 |
| [secretion](http://amigo.geneontology.org/cgi-bin/amigo/go.cgi?action=query&view=query&query=GO:0046903&search_constraint=terms) | 3 out of 28 genes, 10.7% | 81678, 24413, 24896 | 1 |
| [transmembrane receptor protein tyrosine kinase signaling pathway](http://amigo.geneontology.org/cgi-bin/amigo/go.cgi?action=query&view=query&query=GO:0007169&search_constraint=terms) | 3 out of 28 genes, 10.7% | 81678, 306141, 94268 | 1 |
| [nitrogen compound transport](http://amigo.geneontology.org/cgi-bin/amigo/go.cgi?action=query&view=query&query=GO:0071705&search_constraint=terms) | 3 out of 28 genes, 10.7% | 81678, 24413, 24896 | 1 |
| [response to peptide hormone stimulus](http://amigo.geneontology.org/cgi-bin/amigo/go.cgi?action=query&view=query&query=GO:0043434&search_constraint=terms) | 2 out of 28 genes, 7.1% | 24413, 24896 | 1 |
| [response to peptide](http://amigo.geneontology.org/cgi-bin/amigo/go.cgi?action=query&view=query&query=GO:1901652&search_constraint=terms) | 2 out of 28 genes, 7.1% | 24413, 24896 | 1 |
| [energy derivation by oxidation of organic compounds](http://amigo.geneontology.org/cgi-bin/amigo/go.cgi?action=query&view=query&query=GO:0015980&search_constraint=terms) | 2 out of 28 genes, 7.1% | 81678, 24896 | 1 |
| [positive regulation of intracellular protein kinase cascade](http://amigo.geneontology.org/cgi-bin/amigo/go.cgi?action=query&view=query&query=GO:0010740&search_constraint=terms) | 2 out of 28 genes, 7.1% | 313022, 306141 | 1 |
| [growth](http://amigo.geneontology.org/cgi-bin/amigo/go.cgi?action=query&view=query&query=GO:0040007&search_constraint=terms) | 3 out of 28 genes, 10.7% | 306141, 24896, 25177 | 1 |
| [nervous system development](http://amigo.geneontology.org/cgi-bin/amigo/go.cgi?action=query&view=query&query=GO:0007399&search_constraint=terms) | 5 out of 28 genes, 17.9% | 299907, 308022, 29589, 24413, 94268 | 1 |
| [regulation of MAPK cascade](http://amigo.geneontology.org/cgi-bin/amigo/go.cgi?action=query&view=query&query=GO:0043408&search_constraint=terms) | 2 out of 28 genes, 7.1% | 313022, 306141 | 1 |
| [cell differentiation](http://amigo.geneontology.org/cgi-bin/amigo/go.cgi?action=query&view=query&query=GO:0030154&search_constraint=terms) | 6 out of 28 genes, 21.4% | 308022, 29589, 363256, 85251, 369017, 94268 | 1 |
| [positive regulation of protein phosphorylation](http://amigo.geneontology.org/cgi-bin/amigo/go.cgi?action=query&view=query&query=GO:0001934&search_constraint=terms) | 2 out of 28 genes, 7.1% | 313022, 306141 | 1 |
| [positive regulation of phosphorylation](http://amigo.geneontology.org/cgi-bin/amigo/go.cgi?action=query&view=query&query=GO:0042327&search_constraint=terms) | 2 out of 28 genes, 7.1% | 313022, 306141 | 1 |
| [regulation of protein kinase activity](http://amigo.geneontology.org/cgi-bin/amigo/go.cgi?action=query&view=query&query=GO:0045859&search_constraint=terms) | 2 out of 28 genes, 7.1% | 313022, 306141 | 1 |
| [positive regulation of protein modification process](http://amigo.geneontology.org/cgi-bin/amigo/go.cgi?action=query&view=query&query=GO:0031401&search_constraint=terms) | 2 out of 28 genes, 7.1% | 313022, 306141 | 1 |
| [positive regulation of cellular protein metabolic process](http://amigo.geneontology.org/cgi-bin/amigo/go.cgi?action=query&view=query&query=GO:0032270&search_constraint=terms) | 2 out of 28 genes, 7.1% | 313022, 306141 | 1 |
| [sensory organ development](http://amigo.geneontology.org/cgi-bin/amigo/go.cgi?action=query&view=query&query=GO:0007423&search_constraint=terms) | 2 out of 28 genes, 7.1% | 308022, 306141 | 1 |
| [positive regulation of phosphorus metabolic process](http://amigo.geneontology.org/cgi-bin/amigo/go.cgi?action=query&view=query&query=GO:0010562&search_constraint=terms) | 2 out of 28 genes, 7.1% | 313022, 306141 | 1 |
| [positive regulation of phosphate metabolic process](http://amigo.geneontology.org/cgi-bin/amigo/go.cgi?action=query&view=query&query=GO:0045937&search_constraint=terms) | 2 out of 28 genes, 7.1% | 313022, 306141 | 1 |
| [central nervous system development](http://amigo.geneontology.org/cgi-bin/amigo/go.cgi?action=query&view=query&query=GO:0007417&search_constraint=terms) | 3 out of 28 genes, 10.7% | 299907, 29589, 24413 | 1 |
| [cell proliferation](http://amigo.geneontology.org/cgi-bin/amigo/go.cgi?action=query&view=query&query=GO:0008283&search_constraint=terms) | 4 out of 28 genes, 14.3% | 363256, 85251, 24413, 306141 | 1 |
| [cellular response to organic substance](http://amigo.geneontology.org/cgi-bin/amigo/go.cgi?action=query&view=query&query=GO:0071310&search_constraint=terms) | 2 out of 28 genes, 7.1% | 24413, 306141 | 1 |
| [positive regulation of protein metabolic process](http://amigo.geneontology.org/cgi-bin/amigo/go.cgi?action=query&view=query&query=GO:0051247&search_constraint=terms) | 2 out of 28 genes, 7.1% | 313022, 306141 | 1 |
| [generation of precursor metabolites and energy](http://amigo.geneontology.org/cgi-bin/amigo/go.cgi?action=query&view=query&query=GO:0006091&search_constraint=terms) | 2 out of 28 genes, 7.1% | 81678, 24896 | 1 |
| [tube development](http://amigo.geneontology.org/cgi-bin/amigo/go.cgi?action=query&view=query&query=GO:0035295&search_constraint=terms) | 2 out of 28 genes, 7.1% | 24413, 306141 | 1 |
| [oxidation-reduction process](http://amigo.geneontology.org/cgi-bin/amigo/go.cgi?action=query&view=query&query=GO:0055114&search_constraint=terms) | 2 out of 28 genes, 7.1% | 81678, 24896 | 1 |
| [cell-cell signaling](http://amigo.geneontology.org/cgi-bin/amigo/go.cgi?action=query&view=query&query=GO:0007267&search_constraint=terms) | 3 out of 28 genes, 10.7% | 81678, 24413, 24896 | 1 |
| [regulation of kinase activity](http://amigo.geneontology.org/cgi-bin/amigo/go.cgi?action=query&view=query&query=GO:0043549&search_constraint=terms) | 2 out of 28 genes, 7.1% | 313022, 306141 | 1 |
| [regulation of transferase activity](http://amigo.geneontology.org/cgi-bin/amigo/go.cgi?action=query&view=query&query=GO:0051338&search_constraint=terms) | 2 out of 28 genes, 7.1% | 313022, 306141 | 1 |
| [embryo development](http://amigo.geneontology.org/cgi-bin/amigo/go.cgi?action=query&view=query&query=GO:0009790&search_constraint=terms) | 3 out of 28 genes, 10.7% | 299907, 306141, 24896 | 1 |
| [positive regulation of catalytic activity](http://amigo.geneontology.org/cgi-bin/amigo/go.cgi?action=query&view=query&query=GO:0043085&search_constraint=terms) | 2 out of 28 genes, 7.1% | 81678, 313022 | 1 |
| [positive regulation of signal transduction](http://amigo.geneontology.org/cgi-bin/amigo/go.cgi?action=query&view=query&query=GO:0009967&search_constraint=terms) | 2 out of 28 genes, 7.1% | 313022, 306141 | 1 |
| [ion transport](http://amigo.geneontology.org/cgi-bin/amigo/go.cgi?action=query&view=query&query=GO:0006811&search_constraint=terms) | 4 out of 28 genes, 14.3% | 81678, 308022, 24413, 79219 | 1 |
| [response to organic nitrogen](http://amigo.geneontology.org/cgi-bin/amigo/go.cgi?action=query&view=query&query=GO:0010243&search_constraint=terms) | 2 out of 28 genes, 7.1% | 24413, 24896 | 1 |
| [positive regulation of signaling](http://amigo.geneontology.org/cgi-bin/amigo/go.cgi?action=query&view=query&query=GO:0023056&search_constraint=terms) | 2 out of 28 genes, 7.1% | 313022, 306141 | 1 |
| [positive regulation of cell communication](http://amigo.geneontology.org/cgi-bin/amigo/go.cgi?action=query&view=query&query=GO:0010647&search_constraint=terms) | 2 out of 28 genes, 7.1% | 313022, 306141 | 1 |
| [regulation of cellular metabolic process](http://amigo.geneontology.org/cgi-bin/amigo/go.cgi?action=query&view=query&query=GO:0031323&search_constraint=terms) | 7 out of 28 genes, 25.0% | 313022, 29714, 306141, 313596, 362456, 64553, 94268 | 1 |
| [epithelium development](http://amigo.geneontology.org/cgi-bin/amigo/go.cgi?action=query&view=query&query=GO:0060429&search_constraint=terms) | 2 out of 28 genes, 7.1% | 85251, 24413 | 1 |
| [single-organism cellular process](http://amigo.geneontology.org/cgi-bin/amigo/go.cgi?action=query&view=query&query=GO:0044763&search_constraint=terms) | 18 out of 28 genes, 64.3% | 363256, 24896, 25177, 81678, 306141, 315059, 303901, 287709, 24413, 369017, 297604, 300653, 29589, 308022, 297096, 85251, 293976, 94268 | 1 |
| [positive regulation of macromolecule metabolic process](http://amigo.geneontology.org/cgi-bin/amigo/go.cgi?action=query&view=query&query=GO:0010604&search_constraint=terms) | 2 out of 28 genes, 7.1% | 313022, 306141 | 1 |
| [response to nitrogen compound](http://amigo.geneontology.org/cgi-bin/amigo/go.cgi?action=query&view=query&query=GO:1901698&search_constraint=terms) | 2 out of 28 genes, 7.1% | 24413, 24896 | 1 |
| [hemostasis](http://amigo.geneontology.org/cgi-bin/amigo/go.cgi?action=query&view=query&query=GO:0007599&search_constraint=terms) | 2 out of 28 genes, 7.1% | 81678, 24896 | 1 |
| [cellular response to chemical stimulus](http://amigo.geneontology.org/cgi-bin/amigo/go.cgi?action=query&view=query&query=GO:0070887&search_constraint=terms) | 2 out of 28 genes, 7.1% | 24413, 306141 | 1 |
| [regulation of cellular process](http://amigo.geneontology.org/cgi-bin/amigo/go.cgi?action=query&view=query&query=GO:0050794&search_constraint=terms) | 14 out of 28 genes, 50.0% | 313022, 24413, 29714, 297604, 313596, 369017, 362456, 64553, 24896, 81678, 85251, 306141, 94268, 287709 | 1 |
| [regulation of metabolic process](http://amigo.geneontology.org/cgi-bin/amigo/go.cgi?action=query&view=query&query=GO:0019222&search_constraint=terms) | 8 out of 28 genes, 28.6% | 81678, 313022, 29714, 306141, 313596, 362456, 64553, 94268 | 1 |
| [ribonucleoside triphosphate catabolic process](http://amigo.geneontology.org/cgi-bin/amigo/go.cgi?action=query&view=query&query=GO:0009203&search_constraint=terms) | 2 out of 28 genes, 7.1% | 287709, 24896 | 1 |
| [purine ribonucleoside triphosphate catabolic process](http://amigo.geneontology.org/cgi-bin/amigo/go.cgi?action=query&view=query&query=GO:0009207&search_constraint=terms) | 2 out of 28 genes, 7.1% | 287709, 24896 | 1 |
| [regulation of nucleobase-containing compound metabolic process](http://amigo.geneontology.org/cgi-bin/amigo/go.cgi?action=query&view=query&query=GO:0019219&search_constraint=terms) | 5 out of 28 genes, 17.9% | 29714, 306141, 313596, 362456, 94268 | 1 |
| [purine nucleoside triphosphate catabolic process](http://amigo.geneontology.org/cgi-bin/amigo/go.cgi?action=query&view=query&query=GO:0009146&search_constraint=terms) | 2 out of 28 genes, 7.1% | 287709, 24896 | 1 |
| [nucleoside triphosphate catabolic process](http://amigo.geneontology.org/cgi-bin/amigo/go.cgi?action=query&view=query&query=GO:0009143&search_constraint=terms) | 2 out of 28 genes, 7.1% | 287709, 24896 | 1 |
| [regulation of nitrogen compound metabolic process](http://amigo.geneontology.org/cgi-bin/amigo/go.cgi?action=query&view=query&query=GO:0051171&search_constraint=terms) | 5 out of 28 genes, 17.9% | 29714, 306141, 313596, 362456, 94268 | 1 |
| [intracellular protein kinase cascade](http://amigo.geneontology.org/cgi-bin/amigo/go.cgi?action=query&view=query&query=GO:0007243&search_constraint=terms) | 2 out of 28 genes, 7.1% | 297604, 94268 | 1 |
| [regulation of protein phosphorylation](http://amigo.geneontology.org/cgi-bin/amigo/go.cgi?action=query&view=query&query=GO:0001932&search_constraint=terms) | 2 out of 28 genes, 7.1% | 313022, 306141 | 1 |
| [positive regulation of cellular metabolic process](http://amigo.geneontology.org/cgi-bin/amigo/go.cgi?action=query&view=query&query=GO:0031325&search_constraint=terms) | 2 out of 28 genes, 7.1% | 313022, 306141 | 1 |
| [anatomical structure formation involved in morphogenesis](http://amigo.geneontology.org/cgi-bin/amigo/go.cgi?action=query&view=query&query=GO:0048646&search_constraint=terms) | 2 out of 28 genes, 7.1% | 85251, 94268 | 1 |
| [nucleoside phosphate catabolic process](http://amigo.geneontology.org/cgi-bin/amigo/go.cgi?action=query&view=query&query=GO:1901292&search_constraint=terms) | 2 out of 28 genes, 7.1% | 287709, 24896 | 1 |
| [organophosphate catabolic process](http://amigo.geneontology.org/cgi-bin/amigo/go.cgi?action=query&view=query&query=GO:0046434&search_constraint=terms) | 2 out of 28 genes, 7.1% | 287709, 24896 | 1 |
| [purine ribonucleoside triphosphate metabolic process](http://amigo.geneontology.org/cgi-bin/amigo/go.cgi?action=query&view=query&query=GO:0009205&search_constraint=terms) | 2 out of 28 genes, 7.1% | 287709, 24896 | 1 |
| [regulation of body fluid levels](http://amigo.geneontology.org/cgi-bin/amigo/go.cgi?action=query&view=query&query=GO:0050878&search_constraint=terms) | 2 out of 28 genes, 7.1% | 81678, 24896 | 1 |
| [ribonucleoside triphosphate metabolic process](http://amigo.geneontology.org/cgi-bin/amigo/go.cgi?action=query&view=query&query=GO:0009199&search_constraint=terms) | 2 out of 28 genes, 7.1% | 287709, 24896 | 1 |
| [generation of neurons](http://amigo.geneontology.org/cgi-bin/amigo/go.cgi?action=query&view=query&query=GO:0048699&search_constraint=terms) | 2 out of 28 genes, 7.1% | 308022, 29589 | 1 |
| [phosphate-containing compound metabolic process](http://amigo.geneontology.org/cgi-bin/amigo/go.cgi?action=query&view=query&query=GO:0006796&search_constraint=terms) | 5 out of 28 genes, 17.9% | 313022, 301056, 29714, 287709, 24896 | 1 |
| [purine nucleoside triphosphate metabolic process](http://amigo.geneontology.org/cgi-bin/amigo/go.cgi?action=query&view=query&query=GO:0009144&search_constraint=terms) | 2 out of 28 genes, 7.1% | 287709, 24896 | 1 |
| [single-organism process](http://amigo.geneontology.org/cgi-bin/amigo/go.cgi?action=query&view=query&query=GO:0044699&search_constraint=terms) | 20 out of 28 genes, 71.4% | 363256, 24896, 25177, 81678, 306141, 315059, 303901, 287709, 24413, 369017, 79219, 297604, 299907, 300653, 297096, 29589, 308022, 85251, 293976, 94268 | 1 |
| [nucleoside triphosphate metabolic process](http://amigo.geneontology.org/cgi-bin/amigo/go.cgi?action=query&view=query&query=GO:0009141&search_constraint=terms) | 2 out of 28 genes, 7.1% | 287709, 24896 | 1 |
| [response to abiotic stimulus](http://amigo.geneontology.org/cgi-bin/amigo/go.cgi?action=query&view=query&query=GO:0009628&search_constraint=terms) | 2 out of 28 genes, 7.1% | 24413, 297604 | 1 |
| [positive regulation of molecular function](http://amigo.geneontology.org/cgi-bin/amigo/go.cgi?action=query&view=query&query=GO:0044093&search_constraint=terms) | 2 out of 28 genes, 7.1% | 81678, 313022 | 1 |
| [negative regulation of cellular process](http://amigo.geneontology.org/cgi-bin/amigo/go.cgi?action=query&view=query&query=GO:0048523&search_constraint=terms) | 4 out of 28 genes, 14.3% | 24413, 306141, 369017, 94268 | 1 |
| [programmed cell death](http://amigo.geneontology.org/cgi-bin/amigo/go.cgi?action=query&view=query&query=GO:0012501&search_constraint=terms) | 3 out of 28 genes, 10.7% | 85251, 297604, 306141 | 1 |
| [cellular process](http://amigo.geneontology.org/cgi-bin/amigo/go.cgi?action=query&view=query&query=GO:0009987&search_constraint=terms) | 23 out of 28 genes, 82.1% | 313022, 363256, 64445, 24896, 25177, 81678, 301056, 306141, 315059, 303901, 287709, 29714, 24413, 297604, 369017, 300653, 299907, 297096, 308022, 29589, 85251, 293976, 94268 | 1 |
| [regulation of primary metabolic process](http://amigo.geneontology.org/cgi-bin/amigo/go.cgi?action=query&view=query&query=GO:0080090&search_constraint=terms) | 6 out of 28 genes, 21.4% | 313022, 29714, 306141, 313596, 362456, 94268 | 1 |
| [phosphorus metabolic process](http://amigo.geneontology.org/cgi-bin/amigo/go.cgi?action=query&view=query&query=GO:0006793&search_constraint=terms) | 5 out of 28 genes, 17.9% | 313022, 301056, 29714, 287709, 24896 | 1 |
| [cell death](http://amigo.geneontology.org/cgi-bin/amigo/go.cgi?action=query&view=query&query=GO:0008219&search_constraint=terms) | 3 out of 28 genes, 10.7% | 85251, 297604, 306141 | 1 |
| [death](http://amigo.geneontology.org/cgi-bin/amigo/go.cgi?action=query&view=query&query=GO:0016265&search_constraint=terms) | 3 out of 28 genes, 10.7% | 85251, 297604, 306141 | 1 |
| [regulation of phosphorylation](http://amigo.geneontology.org/cgi-bin/amigo/go.cgi?action=query&view=query&query=GO:0042325&search_constraint=terms) | 2 out of 28 genes, 7.1% | 313022, 306141 | 1 |
| [cell activation](http://amigo.geneontology.org/cgi-bin/amigo/go.cgi?action=query&view=query&query=GO:0001775&search_constraint=terms) | 2 out of 28 genes, 7.1% | 81678, 24896 | 1 |
| [neurogenesis](http://amigo.geneontology.org/cgi-bin/amigo/go.cgi?action=query&view=query&query=GO:0022008&search_constraint=terms) | 2 out of 28 genes, 7.1% | 308022, 29589 | 1 |
| [regulation of protein modification process](http://amigo.geneontology.org/cgi-bin/amigo/go.cgi?action=query&view=query&query=GO:0031399&search_constraint=terms) | 2 out of 28 genes, 7.1% | 313022, 306141 | 1 |
| [response to oxygen-containing compound](http://amigo.geneontology.org/cgi-bin/amigo/go.cgi?action=query&view=query&query=GO:1901700&search_constraint=terms) | 2 out of 28 genes, 7.1% | 24413, 24896 | 1 |
| [organ morphogenesis](http://amigo.geneontology.org/cgi-bin/amigo/go.cgi?action=query&view=query&query=GO:0009887&search_constraint=terms) | 2 out of 28 genes, 7.1% | 306141, 24896 | 1 |
| [macromolecule modification](http://amigo.geneontology.org/cgi-bin/amigo/go.cgi?action=query&view=query&query=GO:0043412&search_constraint=terms) | 5 out of 28 genes, 17.9% | 313022, 363256, 29714, 64445, 24896 | 1 |
| [regulation of signal transduction](http://amigo.geneontology.org/cgi-bin/amigo/go.cgi?action=query&view=query&query=GO:0009966&search_constraint=terms) | 3 out of 28 genes, 10.7% | 313022, 306141, 64553 | 1 |
| [establishment of localization in cell](http://amigo.geneontology.org/cgi-bin/amigo/go.cgi?action=query&view=query&query=GO:0051649&search_constraint=terms) | 3 out of 28 genes, 10.7% | 81678, 24413, 24896 | 1 |
| [positive regulation of metabolic process](http://amigo.geneontology.org/cgi-bin/amigo/go.cgi?action=query&view=query&query=GO:0009893&search_constraint=terms) | 2 out of 28 genes, 7.1% | 313022, 306141 | 1 |
| [regulation of biological process](http://amigo.geneontology.org/cgi-bin/amigo/go.cgi?action=query&view=query&query=GO:0050789&search_constraint=terms) | 14 out of 28 genes, 50.0% | 313022, 24413, 29714, 297604, 313596, 369017, 362456, 64553, 24896, 81678, 85251, 306141, 94268, 287709 | 1 |
| [positive regulation of response to stimulus](http://amigo.geneontology.org/cgi-bin/amigo/go.cgi?action=query&view=query&query=GO:0048584&search_constraint=terms) | 2 out of 28 genes, 7.1% | 313022, 306141 | 1 |
| [transport](http://amigo.geneontology.org/cgi-bin/amigo/go.cgi?action=query&view=query&query=GO:0006810&search_constraint=terms) | 7 out of 28 genes, 25.0% | 81678, 297096, 308022, 24413, 79219, 287709, 24896 | 1 |
| [regulation of cellular protein metabolic process](http://amigo.geneontology.org/cgi-bin/amigo/go.cgi?action=query&view=query&query=GO:0032268&search_constraint=terms) | 2 out of 28 genes, 7.1% | 313022, 306141 | 1 |
| [apoptotic process](http://amigo.geneontology.org/cgi-bin/amigo/go.cgi?action=query&view=query&query=GO:0006915&search_constraint=terms) | 2 out of 28 genes, 7.1% | 85251, 306141 | 1 |
| [nucleobase-containing compound catabolic process](http://amigo.geneontology.org/cgi-bin/amigo/go.cgi?action=query&view=query&query=GO:0034655&search_constraint=terms) | 2 out of 28 genes, 7.1% | 287709, 24896 | 1 |
| [response to endogenous stimulus](http://amigo.geneontology.org/cgi-bin/amigo/go.cgi?action=query&view=query&query=GO:0009719&search_constraint=terms) | 3 out of 28 genes, 10.7% | 24413, 306141, 24896 | 1 |
| [positive regulation of cellular process](http://amigo.geneontology.org/cgi-bin/amigo/go.cgi?action=query&view=query&query=GO:0048522&search_constraint=terms) | 4 out of 28 genes, 14.3% | 313022, 24413, 306141, 24896 | 1 |
| [aromatic compound catabolic process](http://amigo.geneontology.org/cgi-bin/amigo/go.cgi?action=query&view=query&query=GO:0019439&search_constraint=terms) | 2 out of 28 genes, 7.1% | 287709, 24896 | 1 |
| [heterocycle catabolic process](http://amigo.geneontology.org/cgi-bin/amigo/go.cgi?action=query&view=query&query=GO:0046700&search_constraint=terms) | 2 out of 28 genes, 7.1% | 287709, 24896 | 1 |
| [cellular nitrogen compound catabolic process](http://amigo.geneontology.org/cgi-bin/amigo/go.cgi?action=query&view=query&query=GO:0044270&search_constraint=terms) | 2 out of 28 genes, 7.1% | 287709, 24896 | 1 |
| [intracellular signal transduction](http://amigo.geneontology.org/cgi-bin/amigo/go.cgi?action=query&view=query&query=GO:0035556&search_constraint=terms) | 3 out of 28 genes, 10.7% | 297604, 94268, 287709 | 1 |
| [organic cyclic compound catabolic process](http://amigo.geneontology.org/cgi-bin/amigo/go.cgi?action=query&view=query&query=GO:1901361&search_constraint=terms) | 2 out of 28 genes, 7.1% | 287709, 24896 | 1 |
| [negative regulation of biological process](http://amigo.geneontology.org/cgi-bin/amigo/go.cgi?action=query&view=query&query=GO:0048519&search_constraint=terms) | 4 out of 28 genes, 14.3% | 24413, 306141, 369017, 94268 | 1 |
| [regulation of cell differentiation](http://amigo.geneontology.org/cgi-bin/amigo/go.cgi?action=query&view=query&query=GO:0045595&search_constraint=terms) | 2 out of 28 genes, 7.1% | 94268, 24896 | 1 |
| [cellular localization](http://amigo.geneontology.org/cgi-bin/amigo/go.cgi?action=query&view=query&query=GO:0051641&search_constraint=terms) | 3 out of 28 genes, 10.7% | 81678, 24413, 24896 | 1 |
| [cell development](http://amigo.geneontology.org/cgi-bin/amigo/go.cgi?action=query&view=query&query=GO:0048468&search_constraint=terms) | 2 out of 28 genes, 7.1% | 85251, 94268 | 1 |
| [single-organism transport](http://amigo.geneontology.org/cgi-bin/amigo/go.cgi?action=query&view=query&query=GO:0044765&search_constraint=terms) | 5 out of 28 genes, 17.9% | 81678, 308022, 24413, 79219, 24896 | 1 |
| [cellular component organization](http://amigo.geneontology.org/cgi-bin/amigo/go.cgi?action=query&view=query&query=GO:0016043&search_constraint=terms) | 6 out of 28 genes, 21.4% | 297096, 85251, 24413, 369017, 94268, 287709 | 1 |
| [organic cyclic compound biosynthetic process](http://amigo.geneontology.org/cgi-bin/amigo/go.cgi?action=query&view=query&query=GO:1901362&search_constraint=terms) | 3 out of 28 genes, 10.7% | 29589, 24413, 293976 | 1 |
| [regulation of protein metabolic process](http://amigo.geneontology.org/cgi-bin/amigo/go.cgi?action=query&view=query&query=GO:0051246&search_constraint=terms) | 2 out of 28 genes, 7.1% | 313022, 306141 | 1 |
| [establishment of localization](http://amigo.geneontology.org/cgi-bin/amigo/go.cgi?action=query&view=query&query=GO:0051234&search_constraint=terms) | 7 out of 28 genes, 25.0% | 81678, 297096, 308022, 24413, 79219, 287709, 24896 | 1 |
| [regulation of transcription from RNA polymerase II promoter](http://amigo.geneontology.org/cgi-bin/amigo/go.cgi?action=query&view=query&query=GO:0006357&search_constraint=terms) | 2 out of 28 genes, 7.1% | 313596, 94268 | 1 |
| [organic substance transport](http://amigo.geneontology.org/cgi-bin/amigo/go.cgi?action=query&view=query&query=GO:0071702&search_constraint=terms) | 3 out of 28 genes, 10.7% | 81678, 24413, 24896 | 1 |
| [nucleoside phosphate metabolic process](http://amigo.geneontology.org/cgi-bin/amigo/go.cgi?action=query&view=query&query=GO:0006753&search_constraint=terms) | 2 out of 28 genes, 7.1% | 287709, 24896 | 1 |
| [localization](http://amigo.geneontology.org/cgi-bin/amigo/go.cgi?action=query&view=query&query=GO:0051179&search_constraint=terms) | 8 out of 28 genes, 28.6% | 24413, 79219, 24896, 81678, 308022, 297096, 85251, 287709 | 1 |
| [signaling](http://amigo.geneontology.org/cgi-bin/amigo/go.cgi?action=query&view=query&query=GO:0023052&search_constraint=terms) | 11 out of 28 genes, 39.3% | 24413, 29714, 297604, 79219, 24896, 299907, 81678, 306141, 303901, 94268, 287709 | 1 |
| [regulation of signaling](http://amigo.geneontology.org/cgi-bin/amigo/go.cgi?action=query&view=query&query=GO:0023051&search_constraint=terms) | 3 out of 28 genes, 10.7% | 313022, 306141, 64553 | 1 |
| [regulation of cell communication](http://amigo.geneontology.org/cgi-bin/amigo/go.cgi?action=query&view=query&query=GO:0010646&search_constraint=terms) | 3 out of 28 genes, 10.7% | 313022, 306141, 64553 | 1 |
| [nucleobase-containing small molecule metabolic process](http://amigo.geneontology.org/cgi-bin/amigo/go.cgi?action=query&view=query&query=GO:0055086&search_constraint=terms) | 2 out of 28 genes, 7.1% | 287709, 24896 | 1 |
| [cellular component organization or biogenesis](http://amigo.geneontology.org/cgi-bin/amigo/go.cgi?action=query&view=query&query=GO:0071840&search_constraint=terms) | 6 out of 28 genes, 21.4% | 297096, 85251, 24413, 369017, 94268, 287709 | 1 |
| [cellular protein modification process](http://amigo.geneontology.org/cgi-bin/amigo/go.cgi?action=query&view=query&query=GO:0006464&search_constraint=terms) | 4 out of 28 genes, 14.3% | 313022, 363256, 29714, 64445 | 1 |
| [protein modification process](http://amigo.geneontology.org/cgi-bin/amigo/go.cgi?action=query&view=query&query=GO:0036211&search_constraint=terms) | 4 out of 28 genes, 14.3% | 313022, 363256, 29714, 64445 | 1 |
| [cell surface receptor signaling pathway](http://amigo.geneontology.org/cgi-bin/amigo/go.cgi?action=query&view=query&query=GO:0007166&search_constraint=terms) | 5 out of 28 genes, 17.9% | 81678, 24413, 306141, 94268, 24896 | 1 |
| [response to hormone stimulus](http://amigo.geneontology.org/cgi-bin/amigo/go.cgi?action=query&view=query&query=GO:0009725&search_constraint=terms) | 2 out of 28 genes, 7.1% | 24413, 24896 | 1 |
| [transcription, DNA-dependent](http://amigo.geneontology.org/cgi-bin/amigo/go.cgi?action=query&view=query&query=GO:0006351&search_constraint=terms) | 2 out of 28 genes, 7.1% | 29589, 293976 | 1 |
| [RNA biosynthetic process](http://amigo.geneontology.org/cgi-bin/amigo/go.cgi?action=query&view=query&query=GO:0032774&search_constraint=terms) | 2 out of 28 genes, 7.1% | 29589, 293976 | 1 |
| [positive regulation of biological process](http://amigo.geneontology.org/cgi-bin/amigo/go.cgi?action=query&view=query&query=GO:0048518&search_constraint=terms) | 4 out of 28 genes, 14.3% | 313022, 24413, 306141, 24896 | 1 |
| [organelle organization](http://amigo.geneontology.org/cgi-bin/amigo/go.cgi?action=query&view=query&query=GO:0006996&search_constraint=terms) | 3 out of 28 genes, 10.7% | 297096, 24413, 287709 | 1 |
| [locomotion](http://amigo.geneontology.org/cgi-bin/amigo/go.cgi?action=query&view=query&query=GO:0040011&search_constraint=terms) | 2 out of 28 genes, 7.1% | 299907, 85251 | 1 |
| [biological regulation](http://amigo.geneontology.org/cgi-bin/amigo/go.cgi?action=query&view=query&query=GO:0065007&search_constraint=terms) | 14 out of 28 genes, 50.0% | 313022, 24413, 29714, 297604, 313596, 369017, 362456, 64553, 24896, 81678, 85251, 306141, 94268, 287709 | 1 |
| [regulation of developmental process](http://amigo.geneontology.org/cgi-bin/amigo/go.cgi?action=query&view=query&query=GO:0050793&search_constraint=terms) | 2 out of 28 genes, 7.1% | 94268, 24896 | 1 |
| [cellular macromolecule metabolic process](http://amigo.geneontology.org/cgi-bin/amigo/go.cgi?action=query&view=query&query=GO:0044260&search_constraint=terms) | 8 out of 28 genes, 28.6% | 299907, 313022, 29589, 363256, 29714, 64445, 293976, 24896 | 1 |
| [regulation of response to stimulus](http://amigo.geneontology.org/cgi-bin/amigo/go.cgi?action=query&view=query&query=GO:0048583&search_constraint=terms) | 3 out of 28 genes, 10.7% | 313022, 306141, 64553 | 1 |
| [signal transduction](http://amigo.geneontology.org/cgi-bin/amigo/go.cgi?action=query&view=query&query=GO:0007165&search_constraint=terms) | 7 out of 28 genes, 25.0% | 81678, 24413, 297604, 306141, 94268, 287709, 24896 | 1 |
| [cellular macromolecule biosynthetic process](http://amigo.geneontology.org/cgi-bin/amigo/go.cgi?action=query&view=query&query=GO:0034645&search_constraint=terms) | 3 out of 28 genes, 10.7% | 299907, 29589, 293976 | 1 |
| [response to external stimulus](http://amigo.geneontology.org/cgi-bin/amigo/go.cgi?action=query&view=query&query=GO:0009605&search_constraint=terms) | 2 out of 28 genes, 7.1% | 299907, 297604 | 1 |
| [organic substance biosynthetic process](http://amigo.geneontology.org/cgi-bin/amigo/go.cgi?action=query&view=query&query=GO:1901576&search_constraint=terms) | 4 out of 28 genes, 14.3% | 299907, 29589, 24413, 293976 | 1 |
| [response to chemical stimulus](http://amigo.geneontology.org/cgi-bin/amigo/go.cgi?action=query&view=query&query=GO:0042221&search_constraint=terms) | 5 out of 28 genes, 17.9% | 299907, 85251, 24413, 306141, 24896 | 1 |
| [macromolecule biosynthetic process](http://amigo.geneontology.org/cgi-bin/amigo/go.cgi?action=query&view=query&query=GO:0009059&search_constraint=terms) | 3 out of 28 genes, 10.7% | 299907, 29589, 293976 | 1 |
| [organophosphate metabolic process](http://amigo.geneontology.org/cgi-bin/amigo/go.cgi?action=query&view=query&query=GO:0019637&search_constraint=terms) | 2 out of 28 genes, 7.1% | 287709, 24896 | 1 |
| [biosynthetic process](http://amigo.geneontology.org/cgi-bin/amigo/go.cgi?action=query&view=query&query=GO:0009058&search_constraint=terms) | 4 out of 28 genes, 14.3% | 299907, 29589, 24413, 293976 | 1 |
| [single-organism metabolic process](http://amigo.geneontology.org/cgi-bin/amigo/go.cgi?action=query&view=query&query=GO:0044710&search_constraint=terms) | 5 out of 28 genes, 17.9% | 81678, 299907, 24413, 287709, 24896 | 1 |
| [macromolecule metabolic process](http://amigo.geneontology.org/cgi-bin/amigo/go.cgi?action=query&view=query&query=GO:0043170&search_constraint=terms) | 9 out of 28 genes, 32.1% | 313022, 363256, 29714, 64445, 24896, 299907, 29589, 306141, 293976 | 1 |
| [regulation of macromolecule metabolic process](http://amigo.geneontology.org/cgi-bin/amigo/go.cgi?action=query&view=query&query=GO:0060255&search_constraint=terms) | 4 out of 28 genes, 14.3% | 313022, 306141, 313596, 94268 | 1 |
| [response to stimulus](http://amigo.geneontology.org/cgi-bin/amigo/go.cgi?action=query&view=query&query=GO:0050896&search_constraint=terms) | 11 out of 28 genes, 39.3% | 24413, 297604, 24896, 299907, 81678, 308022, 29185, 85251, 306141, 94268, 287709 | 1 |
| [nucleobase-containing compound biosynthetic process](http://amigo.geneontology.org/cgi-bin/amigo/go.cgi?action=query&view=query&query=GO:0034654&search_constraint=terms) | 2 out of 28 genes, 7.1% | 29589, 293976 | 1 |
| [reproductive process](http://amigo.geneontology.org/cgi-bin/amigo/go.cgi?action=query&view=query&query=GO:0022414&search_constraint=terms) | 2 out of 28 genes, 7.1% | 293976, 24896 | 1 |
| [aromatic compound biosynthetic process](http://amigo.geneontology.org/cgi-bin/amigo/go.cgi?action=query&view=query&query=GO:0019438&search_constraint=terms) | 2 out of 28 genes, 7.1% | 29589, 293976 | 1 |
| [heterocycle biosynthetic process](http://amigo.geneontology.org/cgi-bin/amigo/go.cgi?action=query&view=query&query=GO:0018130&search_constraint=terms) | 2 out of 28 genes, 7.1% | 29589, 293976 | 1 |
| [cellular catabolic process](http://amigo.geneontology.org/cgi-bin/amigo/go.cgi?action=query&view=query&query=GO:0044248&search_constraint=terms) | 2 out of 28 genes, 7.1% | 287709, 24896 | 1 |
| [single organism signaling](http://amigo.geneontology.org/cgi-bin/amigo/go.cgi?action=query&view=query&query=GO:0044700&search_constraint=terms) | 7 out of 28 genes, 25.0% | 81678, 24413, 297604, 306141, 94268, 287709, 24896 | 1 |
| [cellular response to stimulus](http://amigo.geneontology.org/cgi-bin/amigo/go.cgi?action=query&view=query&query=GO:0051716&search_constraint=terms) | 7 out of 28 genes, 25.0% | 81678, 24413, 297604, 306141, 94268, 287709, 24896 | 1 |
| [cellular nitrogen compound biosynthetic process](http://amigo.geneontology.org/cgi-bin/amigo/go.cgi?action=query&view=query&query=GO:0044271&search_constraint=terms) | 2 out of 28 genes, 7.1% | 29589, 293976 | 1 |
| [reproduction](http://amigo.geneontology.org/cgi-bin/amigo/go.cgi?action=query&view=query&query=GO:0000003&search_constraint=terms) | 2 out of 28 genes, 7.1% | 293976, 24896 | 1 |
| [response to organic substance](http://amigo.geneontology.org/cgi-bin/amigo/go.cgi?action=query&view=query&query=GO:0010033&search_constraint=terms) | 3 out of 28 genes, 10.7% | 24413, 306141, 24896 | 1 |
| [cell communication](http://amigo.geneontology.org/cgi-bin/amigo/go.cgi?action=query&view=query&query=GO:0007154&search_constraint=terms) | 7 out of 28 genes, 25.0% | 81678, 24413, 297604, 306141, 94268, 287709, 24896 | 1 |
| [immune system process](http://amigo.geneontology.org/cgi-bin/amigo/go.cgi?action=query&view=query&query=GO:0002376&search_constraint=terms) | 2 out of 28 genes, 7.1% | 29185, 297604 | 1 |
| [cellular metabolic process](http://amigo.geneontology.org/cgi-bin/amigo/go.cgi?action=query&view=query&query=GO:0044237&search_constraint=terms) | 11 out of 28 genes, 39.3% | 313022, 363256, 29714, 64445, 24896, 299907, 81678, 29589, 301056, 293976, 287709 | 1 |
| [organic substance catabolic process](http://amigo.geneontology.org/cgi-bin/amigo/go.cgi?action=query&view=query&query=GO:1901575&search_constraint=terms) | 2 out of 28 genes, 7.1% | 287709, 24896 | 1 |
| [organic cyclic compound metabolic process](http://amigo.geneontology.org/cgi-bin/amigo/go.cgi?action=query&view=query&query=GO:1901360&search_constraint=terms) | 5 out of 28 genes, 17.9% | 29589, 24413, 293976, 287709, 24896 | 1 |
| [metabolic process](http://amigo.geneontology.org/cgi-bin/amigo/go.cgi?action=query&view=query&query=GO:0008152&search_constraint=terms) | 13 out of 28 genes, 46.4% | 313022, 363256, 24413, 29714, 64445, 24896, 299907, 81678, 29589, 301056, 306141, 293976, 287709 | 1 |
| [protein metabolic process](http://amigo.geneontology.org/cgi-bin/amigo/go.cgi?action=query&view=query&query=GO:0019538&search_constraint=terms) | 5 out of 28 genes, 17.9% | 299907, 313022, 363256, 29714, 64445 | 1 |
| [regulation of transcription, DNA-dependent](http://amigo.geneontology.org/cgi-bin/amigo/go.cgi?action=query&view=query&query=GO:0006355&search_constraint=terms) | 2 out of 28 genes, 7.1% | 313596, 94268 | 1 |
| [cellular protein metabolic process](http://amigo.geneontology.org/cgi-bin/amigo/go.cgi?action=query&view=query&query=GO:0044267&search_constraint=terms) | 4 out of 28 genes, 14.3% | 313022, 363256, 29714, 64445 | 1 |
| [regulation of RNA biosynthetic process](http://amigo.geneontology.org/cgi-bin/amigo/go.cgi?action=query&view=query&query=GO:2001141&search_constraint=terms) | 2 out of 28 genes, 7.1% | 313596, 94268 | 1 |
| [gene expression](http://amigo.geneontology.org/cgi-bin/amigo/go.cgi?action=query&view=query&query=GO:0010467&search_constraint=terms) | 3 out of 28 genes, 10.7% | 29589, 306141, 293976 | 1 |
| [regulation of RNA metabolic process](http://amigo.geneontology.org/cgi-bin/amigo/go.cgi?action=query&view=query&query=GO:0051252&search_constraint=terms) | 2 out of 28 genes, 7.1% | 313596, 94268 | 1 |
| [nucleic acid metabolic process](http://amigo.geneontology.org/cgi-bin/amigo/go.cgi?action=query&view=query&query=GO:0090304&search_constraint=terms) | 3 out of 28 genes, 10.7% | 29589, 293976, 24896 | 1 |
| [catabolic process](http://amigo.geneontology.org/cgi-bin/amigo/go.cgi?action=query&view=query&query=GO:0009056&search_constraint=terms) | 2 out of 28 genes, 7.1% | 287709, 24896 | 1 |
| [small molecule metabolic process](http://amigo.geneontology.org/cgi-bin/amigo/go.cgi?action=query&view=query&query=GO:0044281&search_constraint=terms) | 3 out of 28 genes, 10.7% | 299907, 287709, 24896 | 1 |
| [cellular biosynthetic process](http://amigo.geneontology.org/cgi-bin/amigo/go.cgi?action=query&view=query&query=GO:0044249&search_constraint=terms) | 3 out of 28 genes, 10.7% | 299907, 29589, 293976 | 1 |
| [nucleobase-containing compound metabolic process](http://amigo.geneontology.org/cgi-bin/amigo/go.cgi?action=query&view=query&query=GO:0006139&search_constraint=terms) | 4 out of 28 genes, 14.3% | 29589, 293976, 287709, 24896 | 1 |
| [regulation of cellular macromolecule biosynthetic process](http://amigo.geneontology.org/cgi-bin/amigo/go.cgi?action=query&view=query&query=GO:2000112&search_constraint=terms) | 2 out of 28 genes, 7.1% | 313596, 94268 | 1 |
| [nitrogen compound metabolic process](http://amigo.geneontology.org/cgi-bin/amigo/go.cgi?action=query&view=query&query=GO:0006807&search_constraint=terms) | 5 out of 28 genes, 17.9% | 299907, 29589, 293976, 287709, 24896 | 1 |
| [organic substance metabolic process](http://amigo.geneontology.org/cgi-bin/amigo/go.cgi?action=query&view=query&query=GO:0071704&search_constraint=terms) | 11 out of 28 genes, 39.3% | 313022, 363256, 24413, 29714, 64445, 24896, 299907, 29589, 306141, 293976, 287709 | 1 |
| [RNA metabolic process](http://amigo.geneontology.org/cgi-bin/amigo/go.cgi?action=query&view=query&query=GO:0016070&search_constraint=terms) | 2 out of 28 genes, 7.1% | 29589, 293976 | 1 |
| [heterocycle metabolic process](http://amigo.geneontology.org/cgi-bin/amigo/go.cgi?action=query&view=query&query=GO:0046483&search_constraint=terms) | 4 out of 28 genes, 14.3% | 29589, 293976, 287709, 24896 | 1 |
| [regulation of macromolecule biosynthetic process](http://amigo.geneontology.org/cgi-bin/amigo/go.cgi?action=query&view=query&query=GO:0010556&search_constraint=terms) | 2 out of 28 genes, 7.1% | 313596, 94268 | 1 |
| [regulation of biological quality](http://amigo.geneontology.org/cgi-bin/amigo/go.cgi?action=query&view=query&query=GO:0065008&search_constraint=terms) | 3 out of 28 genes, 10.7% | 81678, 24413, 24896 | 1 |
| [primary metabolic process](http://amigo.geneontology.org/cgi-bin/amigo/go.cgi?action=query&view=query&query=GO:0044238&search_constraint=terms) | 10 out of 28 genes, 35.7% | 313022, 363256, 24413, 29714, 64445, 24896, 299907, 29589, 293976, 287709 | 1 |
| [cellular aromatic compound metabolic process](http://amigo.geneontology.org/cgi-bin/amigo/go.cgi?action=query&view=query&query=GO:0006725&search_constraint=terms) | 4 out of 28 genes, 14.3% | 29589, 293976, 287709, 24896 | 1 |
| [regulation of cellular biosynthetic process](http://amigo.geneontology.org/cgi-bin/amigo/go.cgi?action=query&view=query&query=GO:0031326&search_constraint=terms) | 2 out of 28 genes, 7.1% | 313596, 94268 | 1 |
| [regulation of gene expression](http://amigo.geneontology.org/cgi-bin/amigo/go.cgi?action=query&view=query&query=GO:0010468&search_constraint=terms) | 2 out of 28 genes, 7.1% | 313596, 94268 | 1 |
| [cellular nitrogen compound metabolic process](http://amigo.geneontology.org/cgi-bin/amigo/go.cgi?action=query&view=query&query=GO:0034641&search_constraint=terms) | 4 out of 28 genes, 14.3% | 29589, 293976, 287709, 24896 | 1 |
| [regulation of biosynthetic process](http://amigo.geneontology.org/cgi-bin/amigo/go.cgi?action=query&view=query&query=GO:0009889&search_constraint=terms) | 2 out of 28 genes, 7.1% | 313596, 94268 | 1 |
| [response to stress](http://amigo.geneontology.org/cgi-bin/amigo/go.cgi?action=query&view=query&query=GO:0006950&search_constraint=terms) | 2 out of 28 genes, 7.1% | 81678, 85251 | 1 |

**Table S9. The pathways with differentially methylated genes in** promoter.

| **#** | **Pathway** | **DEGs with pathway annotation** | **Differentially expressed genes** | **Pvalue** | **Qvalue** | **Pathway ID** |
| --- | --- | --- | --- | --- | --- | --- |
| 1 | Melanogenesis | 2 (28.57%) | 24896, 366270 | 0.002635675 | 0.06589188 | ko04916 |
| 2 | Vasopressin-regulated water reabsorption | 1 (14.29%) | 24896 | 0.03350676 | 0.12410214 | ko04962 |
| 3 | Taste transduction | 1 (14.29%) | 24896 | 0.04399573 | 0.12410214 | ko04742 |
| 4 | Endocrine and other factor-regulated calcium reabsorption | 1 (14.29%) | 24896 | 0.04816391 | 0.12410214 | ko04961 |
| 5 | Cocaine addiction | 1 (14.29%) | 24896 | 0.04816391 | 0.12410214 | ko05030 |
| 6 | Vibrio cholerae infection | 1 (14.29%) | 24896 | 0.05714157 | 0.12410214 | ko05110 |
| 7 | Long-term depression | 1 (14.29%) | 24896 | 0.05851629 | 0.12410214 | ko04730 |
| 8 | Amphetamine addiction | 1 (14.29%) | 24896 | 0.06672856 | 0.12410214 | ko05031 |
| 9 | Apoptosis | 1 (14.29%) | 83533 | 0.06672856 | 0.12410214 | ko04210 |
| 10 | Gap junction | 1 (14.29%) | 24896 | 0.068772 | 0.12410214 | ko04540 |
| 11 | Morphine addiction | 1 (14.29%) | 24896 | 0.07013217 | 0.12410214 | ko05032 |
| 12 | Bile secretion | 1 (14.29%) | 24896 | 0.07555582 | 0.12410214 | ko04976 |
| 13 | Salivary secretion | 1 (14.29%) | 24896 | 0.07623186 | 0.12410214 | ko04970 |
| 14 | Chagas disease | 1 (14.29%) | 24896 | 0.07893181 | 0.12410214 | ko05142 |
| 15 | Gastric acid secretion | 1 (14.29%) | 24896 | 0.08431142 | 0.12410214 | ko04971 |
| 16 | Amoebiasis | 1 (14.29%) | 24896 | 0.08498197 | 0.12410214 | ko05146 |
| 17 | GnRH signaling pathway | 1 (14.29%) | 24896 | 0.0856521 | 0.12410214 | ko04912 |
| 18 | Pancreatic secretion | 1 (14.29%) | 24896 | 0.09830494 | 0.12410214 | ko04972 |
| 19 | Glutamatergic synapse | 1 (14.29%) | 24896 | 0.09962804 | 0.12410214 | ko04724 |
| 20 | Alcoholism | 1 (14.29%) | 24896 | 0.1016096 | 0.12410214 | ko05034 |
| 21 | Serotonergic synapse | 1 (14.29%) | 24896 | 0.1042458 | 0.12410214 | ko04726 |
| 22 | Dopaminergic synapse | 1 (14.29%) | 24896 | 0.1094984 | 0.12443000 | ko04728 |
| 23 | Dilated cardiomyopathy | 1 (14.29%) | 24896 | 0.1166776 | 0.12682348 | ko05414 |
| 24 | Calcium signaling pathway | 1 (14.29%) | 24896 | 0.1340892 | 0.13919130 | ko04020 |
| 25 | Vascular smooth muscle contraction | 1 (14.29%) | 24896 | 0.1391913 | 0.13919130 | ko04270 |

**Table S10. The pathways with differentially methylated genes in CGI.**

| # | Pathway | DEGs with pathway annotation (31) | Differentially expressed genes | Pvalue | Qvalue | Pathway ID |
| --- | --- | --- | --- | --- | --- | --- |
| 1 | Vasopressin-regulated water reabsorption | 3 (9.68%) | 24896, 287709, 362456 | 0.0004384 | 0.0249876 | ko04962 |
| 2 | Long-term depression | 2 (6.45%) | 24896, 81678 | 0.0287604 | 0.3491249 | ko04730 |
| 3 | Axon guidance | 3 (9.68%) | 94268, 303901, 362945 | 0.0314926 | 0.3491249 | ko04360 |
| 4 | Gap junction | 2 (6.45%) | 24896, 81678 | 0.0390016 | 0.3491249 | ko04540 |
| 5 | Glycosaminoglycan biosynthesis - keratan sulfate | 1 (3.23%) | 64445 | 0.0439422 | 0.3491249 | ko00533 |
| 6 | Salivary secretion | 2 (6.45%) | 24896, 81678 | 0.0472669 | 0.3491249 | ko04970 |
| 7 | Gastric acid secretion | 2 (6.45%) | 24896, 81678 | 0.0569465 | 0.3491249 | ko04971 |
| 8 | Amoebiasis | 2 (6.45%) | 24896, 287709 | 0.0577825 | 0.3491249 | ko05146 |
| 9 | GnRH signaling pathway | 2 (6.45%) | 24896, 81678 | 0.0586228 | 0.3491249 | ko04912 |
| 10 | Pancreatic secretion | 2 (6.45%) | 24896, 81678 | 0.0753711 | 0.3491249 | ko04972 |
| 11 | Glutamatergic synapse | 2 (6.45%) | 24896, 81678 | 0.0772154 | 0.3491249 | ko04724 |
| 12 | Glycosaminoglycan biosynthesis - heparan sulfate | 1 (3.23%) | 299907 | 0.0801146 | 0.3491249 | ko00534 |
| 13 | Serotonergic synapse | 2 (6.45%) | 24896, 81678 | 0.0837835 | 0.3491249 | ko04726 |
| 14 | Dopaminergic synapse | 2 (6.45%) | 24896, 81678 | 0.0914944 | 0.3491249 | ko04728 |
| 15 | Glycosphingolipid biosynthesis - lacto and neolacto series | 1 (3.23%) | 64445 | 0.091875 | 0.3491249 | ko00601 |
| 16 | Intestinal immune network for IgA production | 1 (3.23%) | 297604 | 0.120643 | 0.4060615 | ko04672 |
| 17 | Calcium signaling pathway | 2 (6.45%) | 24896, 81678 | 0.1306637 | 0.4060615 | ko04020 |
| 18 | Vascular smooth muscle contraction | 2 (6.45%) | 24896, 81678 | 0.1393581 | 0.4060615 | ko04270 |
| 19 | Tuberculosis | 2 (6.45%) | 24944, 287709 | 0.1459635 | 0.4060615 | ko05152 |
| 20 | Phagosome | 2 (6.45%) | 24944, 287709 | 0.1481804 | 0.4060615 | ko04145 |
| 21 | Type I diabetes mellitus | 1 (3.23%) | 29714 | 0.1540016 | 0.4060615 | ko04940 |
| 22 | Other types of O-glycan biosynthesis | 1 (3.23%) | 64445 | 0.1567255 | 0.4060615 | ko00514 |
| 23 | Taste transduction | 1 (3.23%) | 24896 | 0.1808619 | 0.4482024 | ko04742 |
| 24 | Endocrine and other factor-regulated calcium reabsorption | 1 (3.23%) | 24896 | 0.19658 | 0.4482024 | ko04961 |
| 25 | Cocaine addiction | 1 (3.23%) | 24896 | 0.19658 | 0.4482024 | ko05030 |
| 26 | Vibrio cholerae infection | 1 (3.23%) | 24896 | 0.2296408 | 0.4506436 | ko05110 |
| 27 | Neuroactive ligand-receptor interaction | 2 (6.45%) | 24413, 79219 | 0.2459114 | 0.4506436 | ko04080 |
| 28 | Long-term potentiation | 1 (3.23%) | 81678 | 0.2493274 | 0.4506436 | ko04720 |
| 29 | Amphetamine addiction | 1 (3.23%) | 24896 | 0.263772 | 0.4506436 | ko05031 |
| 30 | Hematopoietic cell lineage | 1 (3.23%) | 29185 | 0.2685268 | 0.4506436 | ko04640 |
| 31 | Protein digestion and absorption | 1 (3.23%) | 85251 | 0.2732518 | 0.4506436 | ko04974 |
| 32 | Morphine addiction | 1 (3.23%) | 24896 | 0.2756033 | 0.4506436 | ko05032 |
| 33 | Bile secretion | 1 (3.23%) | 24896 | 0.2941516 | 0.4506436 | ko04976 |
| 34 | Retrograde endocannabinoid signaling | 1 (3.23%) | 81678 | 0.2987164 | 0.4506436 | ko04723 |
| 35 | Melanogenesis | 1 (3.23%) | 24896 | 0.300988 | 0.4506436 | ko04916 |
| 36 | Phosphatidylinositol signaling system | 1 (3.23%) | 81678 | 0.30551 | 0.4506436 | ko04070 |
| 37 | Chagas disease (American trypanosomiasis) | 1 (3.23%) | 24896 | 0.30551 | 0.4506436 | ko05142 |
| 38 | Cholinergic synapse | 1 (3.23%) | 81678 | 0.3144692 | 0.4506436 | ko04725 |
| 39 | Adherens junction | 1 (3.23%) | 29616 | 0.3189067 | 0.4506436 | ko04520 |
| 40 | ECM-receptor interaction | 1 (3.23%) | 300653 | 0.3211151 | 0.4506436 | ko04512 |
| 41 | Complement and coagulation cascades | 1 (3.23%) | 300653 | 0.3276986 | 0.4506436 | ko04610 |
| 42 | NF-kappa B signaling pathway | 1 (3.23%) | 297604 | 0.3320532 | 0.4506436 | ko04064 |
| 43 | Oocyte meiosis | 1 (3.23%) | 81678 | 0.3470805 | 0.4600835 | ko04114 |
| 44 | Alcoholism | 1 (3.23%) | 24896 | 0.3781882 | 0.4750996 | ko05034 |
| 45 | Lysosome | 1 (3.23%) | 24944 | 0.3862383 | 0.4750996 | ko04142 |
| 46 | Jak-STAT signaling pathway | 1 (3.23%) | 306141 | 0.3862383 | 0.4750996 | ko04630 |
| 47 | Neurotrophin signaling pathway | 1 (3.23%) | 362456 | 0.3981246 | 0.4750996 | ko04722 |
| 48 | Cell adhesion molecules (CAMs) | 1 (3.23%) | 29616 | 0.4000839 | 0.4750996 | ko04514 |
| 49 | Dilated cardiomyopathy | 1 (3.23%) | 24896 | 0.423119 | 0.4921997 | ko05414 |
| 50 | Alzheimer's disease | 1 (3.23%) | 81678 | 0.4542924 | 0.5178933 | ko05010 |
| 51 | Cytokine-cytokine receptor interaction | 1 (3.23%) | 297604 | 0.5352956 | 0.5982716 | ko04060 |
| 52 | Tight junction | 1 (3.23%) | 64553 | 0.5532837 | 0.6064841 | ko04530 |
| 53 | Focal adhesion | 1 (3.23%) | 300653 | 0.5831543 | 0.627166 | ko04510 |
| 54 | Endocytosis | 1 (3.23%) | 287709 | 0.5966899 | 0.6298393 | ko04144 |
| 55 | HTLV-I infection | 1 (3.23%) | 297604 | 0.616199 | 0.6386062 | ko05166 |
| 56 | MAPK signaling pathway | 1 (3.23%) | 313022 | 0.6372058 | 0.6485845 | ko04010 |
| 57 | Metabolic pathways | 2 (6.45%) | 64445, 299907 | 0.9120982 | 0.9120982 | ko01100 |
